# A Redox‐Tunable Carborane Crown: Toward Highly Selective Electrochemical Lithium Capture

**DOI:** 10.1002/chem.202502902

**Published:** 2025-11-17

**Authors:** Shannon Heinrich, Zongheng Wang, Jitendrasingh Rajpurohit, Ashley Yeow, Roman Dobrovetsky, Lior Sepunaru, Gabriel Ménard

**Affiliations:** ^1^ Department of Chemistry and Biochemistry University of California Santa Barbara California 93106 USA; ^2^ School of Chemistry Raymond and Beverly Sackler Faculty of Exact Sciences Tel Aviv University Tel Aviv 69978 Israel; ^3^ Department of Chemistry University of Calgary 2500 University Drive NW Calgary Alberta T2N 1N4 Canada

**Keywords:** binding constants, carboranes, DLE, electrochemical, lithium

## Abstract

Lithium is a critical element with a projected exponential rise in demand due to its widespread use in battery energy storage. New methods to extract Li^+^, such as through membrane adsorption‐based direct Li^+^ extraction (DLE) technologies, are at various stages of development and aim to separate Li^+^ from brine and even seawater. In this report, we present a fundamentally new class of highly selective Li^+^‐capture agent, the carborane‐crown compound, 1,2‐((6,6,7,7‐Me_4_)14‐crown‐4)‐*ortho*‐carborane (**^14C4^Cb**), which is electrochemically activated for strong, selective Li^+^ binding over Na^+^ and K^+^. This newly synthesized extractant features a redox‐tunable cavity size, giving rise to tunable binding constants for Li^+^ capture, favorable coulombic interactions between the reduced anionic capture agent and the Li^+^ cations, and boasts the benefit of rapid, electrochemically driven capture kinetics. Weak, negligible binding to Li^+^ was observed in the neutral “*closo*” carborane state (**^14C4^Cb**), whereas strong binding was observed in the cage‐opened reduced *nido* state (**^14C4^Cb^2−^**). Equilibrium (*K*) binding constants were measured through experimental and simulated voltammetry, yielding the following log *K*_metal_ (experimental; *simulation*) values: log *K*_Li_ (6.8 ± 0.6; *8.0*), log *K*_Na_ (3.7 ± 0.2; *4.9*), log *K*_K_ (1.7 ± 0.2; *2.2*). Rapid mass transport of Li^+^ to the electrode surface resulted in the simulated value (log *K*_Li_ = 8.0) representing a lower‐limit value for log *K*_Li_ as described herein. The observed strong binding to Li^+^ over Na^+^ and K^+^ is attributed to both the favorable redox‐tunable crown cavity size of the **^14C4^Cb/^14C4^Cb^2−^** couple, combined with strong coulombic interactions in the reduced *nido* state. This platform offers a potential new, rapid, and highly selective technique for Li^+^ capture in next‐generation electrochemical DLE technologies.

## Graphical abstract

Electrochemical reduction transforms a new carborane–crown into a potent 2e^−^ reduced *nido* anion with a redox‐tunable cavity that captures Li⁺ selectively over Na⁺ and K⁺. The coupling of electronic control and size‐matched binding offers a blueprint for selective, rapid lithium recovery for next‐generation direct lithium extraction.

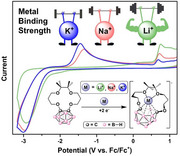

## 1 Introduction

**Figure 1:**
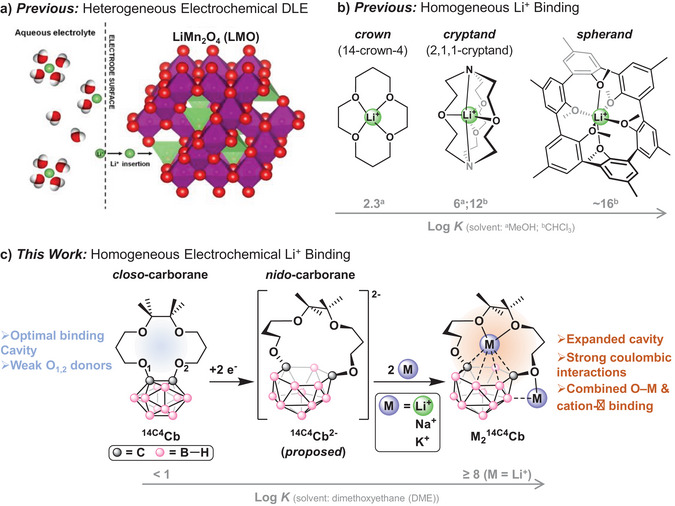
Various approaches to Li^+^ capture. a) Heterogeneous electrochemical DLE using LMO. Image reproduced with permission from Marchini et al.^[^ ^28^ ^]^ b) Homogeneous Li^+^ binding using reported crowns, cryptands, and spherands. The corresponding solvent‐dependent log K values are indicated. c) This work features the electrochemically tunable binding to alkali metals using the redox‐switchable carborane crown compound, ^14C4^Cb.

Lithium is a critical element key to widescale battery energy storage and is needed for our transition from fossil fuels to renewable energy. Current Li^+^ extraction technologies include hard rock mining primarily in Australia and China, as well as salt lake mining using the evaporative lime‐soda process mostly in the “Lithium Triangle” of Chile, Bolivia, and Argentina. However, production from these processes (0.54 Mt Li_2_CO_3_ equivalent (LCE) in 2021) is not expected to meet projected demand (3.3–3.8 Mt LCE by 2030) without the significant development of new Direct Lithium Extraction (DLE) technologies.^[^ ^1^ ^]^ DLE technologies are at various stages of development and all aim to separate Li from brine and even seawater using different techniques, including ion‐exchange resins, biphasic solvent extractions, thermally assisted processes, and electrochemical methods, among others.^[^ ^2^ ^]^ Ion‐exchange resins are the most investigated class of DLE materials and rely on the strong adsorption of Li^+^ cations to, for instance, TiO_2_ or Mn_2_O_4_‐derived materials.^[^ ^3^, ^4^, ^5^, ^6^ ^]^ While these adsorbents are mostly selective for Li^+^, the process is often very slow (i.e., weeks to months), requires an acidic workup that can lead to Mn leaching,^[^ ^7^ ^]^ and often requires further purification of the released Li^+^ concentrate due to the cocapture of other metals.^[^ ^8^ ^]^ Electrochemically, these Mn_2_O_4_ and related LiFePO_4_ adsorbents have been studied as electrode materials (e.g., LMO; Figure 1a), including under flow conditions.^[^ ^7^, ^9^, ^10^, ^11^, ^12^, ^13^ ^]^ The main advantage of using electrochemical methods is to reduce the time required to capture Li^+^. While these techniques show promise, many of these materials suffer from relatively high energy consumption, pH sensitivity, and competing metal binding, as well as metal leaching, which can have negative environmental impacts.^[^ ^7^ ^]^

In contrast to these materials approaches, reported molecular systems have generally featured macrocyclic ligands with size‐selective cation‐binding pockets, which can provide excellent control over Li^+^ capture selectivity. Crown ethers, cryptands, and spherands bind strongly to Lewis acidic metals, such as Li^+^, via oxygen and/or nitrogen‐metal dative bonding (Figure 1b).^[^ ^14^, ^15^, ^16^, ^17^, ^18^, ^19^, ^20^ ^]^ Tuning the selectivity for Li^+^ over Na^+^ and K^+^ by varying the pocket size of the macrocycle is well established. For instance, cavity sizes ranging from 0.6–0.75 Å in crown‐ethers lead to equilibrium log *K* values with Li^+^ of 2.14 ± 0.03 and 2.34 ± 0.06 for 12‐crown‐4 and 14‐crown‐4, respectively.^[^ ^15^ ^]^ In contrast, rigid spherands feature some of the strongest Li^+^ binding constants with log *K* values as high as 16.^[^ ^18^ ^]^ Meanwhile, cryptands such as 2,1,1‐cryptand typically have solvent‐dependent log *K* values in between crowns and spherands (Figure 1b).^[^ ^18^, ^21^ ^]^ While the binding is strongest in spherands and cryptands, removal of Li^+^ requires high temperatures and potential decomposition of the capture agent.^[^ ^14^ ^]^ While crown ethers possess weaker Li^+^ binding than spherands, they are synthetically more accessible, and offer flexibility with a recent report describing their use in Li^+^ selective ion‐exchange resins and membranes.^[^ ^22^ ^]^ Previous works also highlight their use in electrochemical alkali metal cation sensing when tethered to redox‐active moieties, in particular ferrocene.^[^ ^23^, ^24^, ^25^, ^26^, ^27^ ^]^

Herein, we present a fundamentally new class of highly selective electrochemical Li‐capture agent, the carborane‐crown compound, 1,2‐((6,6,7,7‐Me_4_)14‐crown‐4)‐*o*‐carborane (**^14C4^Cb**; Figure 1c). This compound features a redox‐tunable cavity size giving rise to tunable binding constants for capture and release, favorable coulombic interactions between the reduced anionic capture agent and the Li^+^ cations, and the benefit of rapid, electrochemically driven capture kinetics for selective Li^+^ binding versus other alkali metals.^[^ ^9^ ^]^ This new compound builds upon our previous work wherein we used the redox‐switchable properties of a phosphine oxide‐substituted *ortho*‐carborane, 1,2‐(Ph_2_PO)_2_–1,2‐C_2_B_10_H_10_ (**^PO^Cb**), for the selective biphasic or heterogeneous electrochemical capture and release of the uranyl (UO_2_^2+^) ion.^[^ ^29^, ^30^, ^31^, ^32^ ^]^ Reduction of the *closo*‐carborane, **^PO^Cb**, to its 2e^−^ reduced *nido* form (**^PO^Cb^2−^**) resulted in cage C–C bond cleavage and rearrangement, prompting the Ph_2_PO groups to behave as electrochemically switchable ligands with dramatically different *closo‐* to *nido‐*binding affinities for UO_2_^2+^. For instance, selective UO_2_^2+^ capture could be initiated using **^PO^Cb^2−^** with subsequent release occurring upon oxidation to **^PO^Cb**. The increased metal affinity in the *nido‐*form likely arose from the change in the expanded bite angle between the two donor appendages, the increased electron density at the donor sites, as well as favorable coulombic interactions in the reduced state with cationic metal centers.

In this report, we detail the synthesis and use of the new crown‐carborane, **^14C4^Cb**. We suspected that weak binding to Li^+^ may occur in the *closo* form (**^14C4^Cb**) due to the electron‐withdrawing nature of the carborane cage deactivating binding through O1 and O2 (Figure 1c), but that strong binding would prevail in the *nido* form (**^14C4^Cb^2−^**) due to the expanded crown cavity and favorable coulombic interactions with Li^+^. We scrutinized the structural and spectroscopic differences that accompany the chemical and electrochemical reduction of **^14C4^Cb** to **^14C4^Cb^2−^** and estimated the binding affinity of **^14C4^Cb** and **^14C4^Cb^2−^** to Li^+^ Na^+^ and K^+^ through a combination of spectroscopic and electrochemical techniques. As described below, while **^14C4^Cb** displays strong, selective binding to Li^+^, we note that its electrochemical profile is not yet suitable for use in real‐world applications; nonetheless, future synthetic modifications to the crown‐carborane platform may pave the way for future applications. These current results are presented herein.

## 2 Results and Discussion

**Scheme 1:**
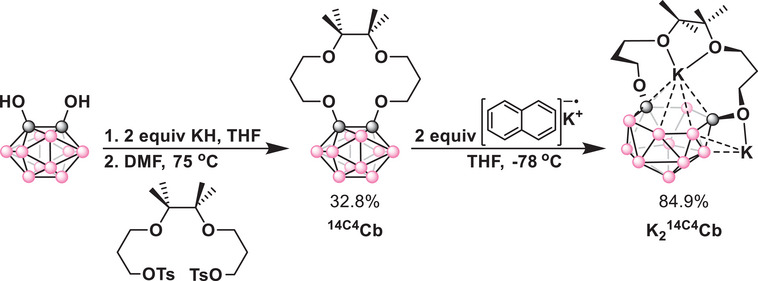
Synthetic scheme for the synthesis of **^14C4^Cb** and **K_2_^14C4^Cb**.

**Figure 2:**
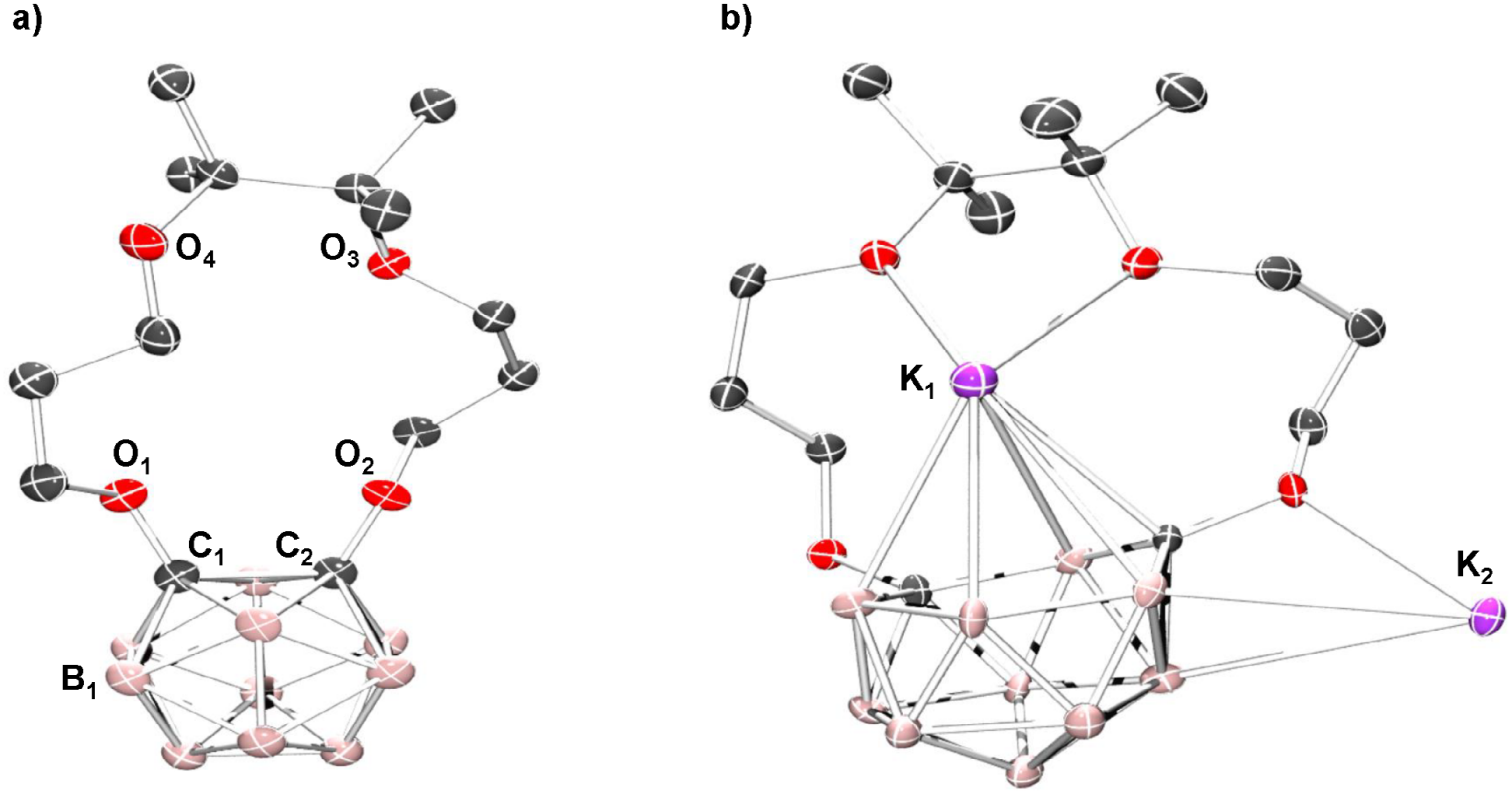
Solid‐state molecular structures of a) **^14C4^Cb** and b) **K_2_^14C4^Cb**. Hydrogen atoms, cocrystallized solvent molecules, and repeating units in b) are omitted for clarity.

We identified **^14C4^Cb** as the target *o‐*carborane crown ether given the higher reported Li^+^ binding constant of 14‐crown‐4 relative to smaller or larger crowns.^[^ ^15^ ^]^ We further raised the Li^+^ selectivity over Na^+^ and K^+^ by replacing the ethylene bridge distal to the *o‐*carborane with a bulkier pinacol bridge. The increased steric repulsion of a pinacol bridge favors smaller cations, Li^+^ over Na^+^.^[^ ^33^, ^34^, ^35^ ^]^ **^14C4^Cb** was synthesized by initial deprotonation of the reported 1,2‐diol‐*o*‐carborane^[^ ^36^ ^]^ with KH in THF followed by cyclization with the reported tosylated polyether, 2,3‐bis(3‐[*p*‐toluenesulfonyloxy]propoxy)‐2,3‐dimethylbutane)^[^ ^37^ ^]^ in DMF at elevated temperatures for 12 hours (Scheme 1). Purification by column chromatography yielded a pure white solid product in 33% isolated yield. Single crystals suitable for X‐ray diffraction (XRD) studies were grown from a saturated solution of the product in diethyl ether at −25 °C. The solid‐state molecular structure of **^14C4^Cb** revealed an elongated carborane C–C bond length of 1.767 Å relative to *ortho*‐carborane (1.624 Å), consistent with O lone pair donation to the C–C σ* bond (Figure 2a).^[^ ^38^ ^]^

We next investigated the reduction of *closo*‐**^14C4^Cb** to its *nido* form by 2e^−^ chemical reduction. To a frozen solution of **^14C4^Cb** in THF, a solution of potassium naphthalenide in THF was added and the frozen solution was allowed to warm to room temperature (Scheme 1). After workup and recrystallization from acetonitrile, the reduced *nido* product, **K_2_^14C4^Cb**, was obtained. Single crystals of **K_2_^14C4^Cb** suitable for XRD studies were obtained from a concentrated acetonitrile solution at −25 °C. The solid‐state molecular structure revealed dramatic structural changes to both the crown and the carborane components upon 2e^−^ reduction (Figure 2b). The C_1_–C_2_ bond was cleaved (2.737 Å) and the boron centers rearranged to form a planar 6‐membered ring with an *η* ^6^ C–B–C–B–B–B configuration. The distance between this 6‐membered ring and the K^+^ center in the cavity is ∼3 Å, reminiscent of similar cation‐π *η* ^6^ bonding interactions observed in potassium metallocene‐crown complexes^[^ ^39^ ^]^ and [C_6_H_6_‐M]^+^ adducts.^[^ ^40^ ^]^ This *η* ^6^ bonding interaction appears to supersede the anticipated formation of O_1_–K and O_2_–K bonds and may be driven by the strong coulombic interaction between the negatively charged cage and the K^+^ cation. The second K^+^ is exogenous to the crown pocket and located between two carboranes, binding a crown oxygen from each, as well as forming an agostic B–H cage and acetonitrile π interactions (Figure S44).

**Figure 3:**
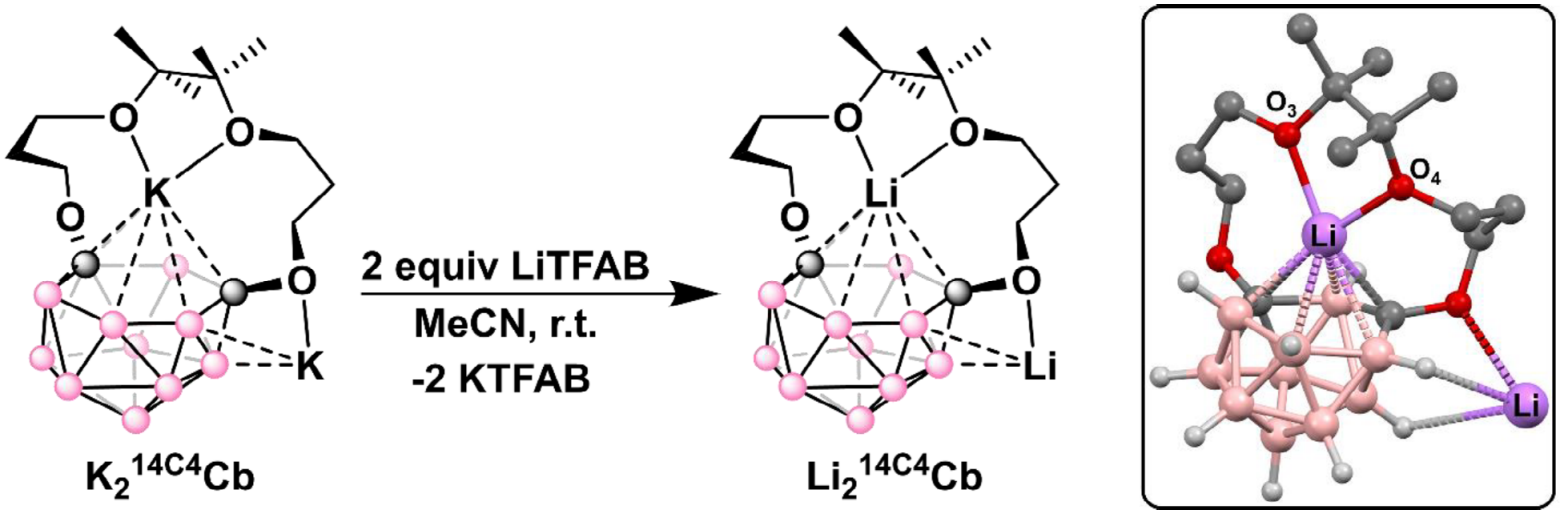
Salt swap of **K_2_^14C4^Cb** with [Li(OEt_2_)_2.5_][TFAB]. Box: DFT‐calculated molecular structure of **Li_2_^14C4^Cb**.

Attempts to isolate the analogous salt, **Li_2_^14C4^Cb**, via reduction using Li naphthalenide failed. We observed many peaks in the ^7^Li and ^11^B NMR spectra, likely due to a difficult‐to‐control stoichiometry given that Li naphthalenide forms a mixture of mono‐ and di‐anionic species.^[^ ^41^ ^]^ Instead, we performed a salt swap by stirring 1 equiv of **K_2_^14C4^Cb** with 2 equiv of lithium tetrakis(pentafluorophenyl)borate ethyl etherate ([Li(OEt_2_)_2.5_][TFAB]) at room temperature in acetonitrile to afford the putative species, **Li_2_^14C4^Cb** (Figure 3). We observed that a white solid partially precipitated out of solution, which we suspected may be [K][TFAB].

Monitoring the reaction by ^11^B NMR spectroscopy, we initially observed at least 12 distinct resonances, likely corresponding to multiple interconverting products. This mixture converted to a single product with 5 different correlated ^11^B resonances within several hours, with some of these resonances being overlapping as determined by integration (Figure S8). The ^7^Li NMR spectrum also converged to a single broadened resonance downfield of free [Li(OEt_2_)_2.5_][TFAB] (Figure S9). The ^1^H NMR spectrum displayed chemically inequivalent methylene resonances (6 total integrating to 2H each) and 2 chemically inequivalent methyl resonances (Figure S7). We suspect that the asymmetry of the ^1^H resonances is likely due to strong, rigid binding between Li^+^ and the crown cavity. Comparatively, the ^1^H NMR spectrum of **K_2_^14C4^Cb** was more symmetric displaying chemically equivalent methylene resonances (3 resonances total integrating to 4H each) and chemically equivalent methyl resonances (1 single resonance integrating to 12H), which, together, we attribute to weaker, flexible K^+^ binding in the crown cavity (vide infra) (Figure S4). Attempts to grow single crystals of **Li_2_^14C4^Cb** suitable for XRD studies failed. In the absence of the molecular structure of **Li_2_^14C4^Cb**, we decided to model it based on the molecular structure obtained for **K_2_^14C4^Cb** using DFT computations. Thus, the fragmental structure of **K_2_^14C4^Cb**, **^mod^K_2_^14C4^Cb**, in which the outer sphere K atom is coordinated to MeOH (see Supporting Information, Section 8 for all DFT structures), was optimized using B3LYP‐D3/def2SVP level of theory,^[^ ^42^ ^]^ while the effect of the solvent (acetonitrile) was taken into account using the continuum solvation model SMD (Solvent Model Density).^[^ ^43^, ^44^, ^45^, ^46^, ^47^ ^]^ Then the K atoms in the optimized structure were replaced by Li, and the fragmental **^mod^Li_2_^14C4^Cb** was calculated using the same level of theory. In contrast to **^mod^K_2_^14C4^Cb,** in which carborane–K binding was obtained (*η* ^6^ plane–K = 2.809 Å), in the optimized **^mod^Li_2_^14C4^Cb** (see Supporting Information, Section 8) the Li atom in the crown pocket has a significantly longer distance to the *η* ^6^ plane (*η* ^6^ plane–Li = 3.494 Å), indicating the very insignificant interaction between carborane and Li. Interestingly, the structure of **^mod^Li_2_^14C4^Cb** without the two coordinating acetonitrile molecules, **^mod1^Li_2_^14C4^Cb** (see Supporting Information, Section 8), was optimized as well, and the loss of the two acetonitrile molecules was found to be slightly exergonic with ΔG = –2.9 kcal mol^−1^. Expectedly, in **^mod1^Li_2_^14C4^Cb,** a tighter crown–Li and carborane–Li binding was obtained with O3–Li (1.965 Å), O4–Li (2.174 Å) and average *η* ^6^ plane–Li (1.922 Å) bond distances. We also optimized the structure of the **^mod1^K_2_^14C4^Cb** (see Supporting Information, Section 8) without the two acetonitrile molecules similarly to the **^mod1^Li_2_^14C4^Cb** and compared these two structures. The optimized **^mod1^Li_2_^14C4^Cb** structure revealed expected tighter crown–Li and carborane–Li binding with O3–Li, O4–Li, and average *η* ^6^ plane–Li bond distances (see above) all being shorter than the corresponding distances in **^mod1^K_2_^14C4^Cb** (O3–K (2.690 Å), O4–K (2.746 Å), *η* ^6^ plane–K (2.732 Å)). These data are further consistent with the smaller ionic radius of Li^+^ versus K^+^. Based on these computational results, we believe that the **Li_2_^14C4^Cb** likely loses two acetonitrile molecules, leading to the complex with a tighter binding of the Li compared to the **K_2_^14C4^Cb**. These results explain the observed asymmetric NMR resonances and are consistent with the proposed tighter binding to Li.

### 2.1 NMR Binding Studies

**Figure 4:**
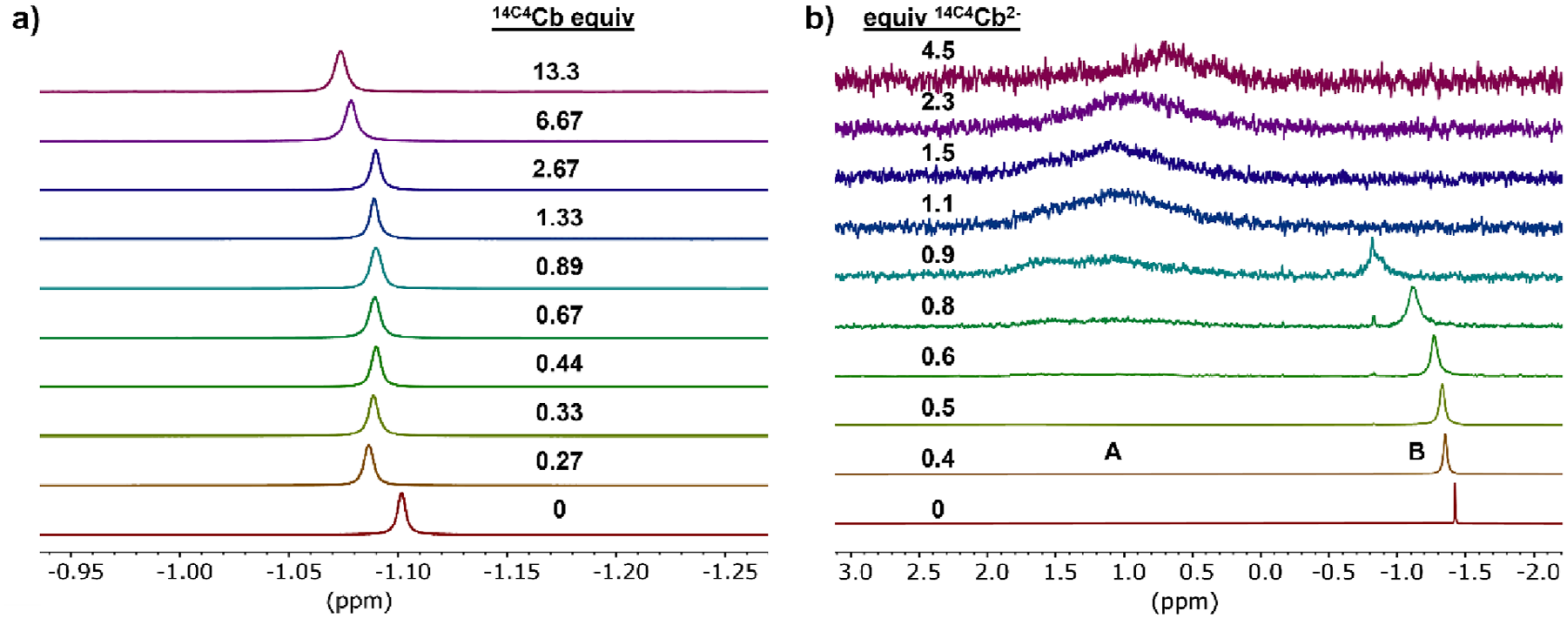
NMR binding studies of *closo* and *nido* **^14C4^Cb** with Li^+^. a) Stacked ^7^Li NMR spectra of [Li][PF_6_] with different equivalents of added **^14C4^Cb** in THF‐d_8_. b) Stacked unlocked ^7^Li NMR spectra of 1 equivalent of [Li(OEt_2_)_2.5_][TFAB] in 0.1 M [Bu_4_N][PF_6_] DME with increasing equivalents of electrochemically‐generated **^14C4^Cb^2−^**.

We evaluated the Li^+^ metal binding behavior of **^14C4^Cb** and the metal‐free *nido* variant, **^14C4^Cb^2−^** (vide infra), using NMR spectroscopy. We acquired the ^1^H NMR spectrum of **^14C4^Cb** in THF‐d_8_ with varying concentrations of Li^+^ added as [Li][PF_6_]. We found the ^1^H NMR resonances corresponding to **^14C4^Cb** remained unaltered with up to 4 equivalents of added Li^+^ (Figure S12). In a complementary experiment, varying concentrations of **^14C4^Cb** were added to a solution of [Li][PF_6_] in THF‐d_8_, and the ^7^Li NMR spectra were recorded. In this case, the resonance corresponding to the solvated Li^+^ shifted marginally (Δ = 0.03 ppm) from 0 to 13.3 equivalents of added **^14C4^Cb** (Figure 4a). The peak full width at half maximum (FWHM) broadened from 0.80 Hz to 1.19 Hz as **^14C4^Cb** was added. This very minor shift and broadening of the ^7^Li resonance suggested that only a weak interaction between Li^+^ and **^14C4^Cb** existed. We attribute this weak coordination—which stands in contrast to the reported 14‐crown‐4^[^ ^15^ ^]^—to the electron‐withdrawing effect of the *ortho*‐carborane cage deactivating the O1 and O2 donors in the macrocycle (Figure 2a). Due to the weak interaction of **^14C4^Cb** with Li^+^ and the corresponding small resonance shifts observed, combined with the already broadened quadrupolar ^7^Li resonance, we were unable to calculate any meaningful binding constants using standard NMR techniques.

To study the binding affinity of the naked *nido* anion, **^14C4^Cb^2−^**, by ^7^Li NMR spectroscopy, we generated it electrochemically. Galvanostatic bulk electrolysis (GBE) was performed on a solution of **^14C4^Cb** in the presence of 0.1 M [Bu_4_N][PF_6_] supporting electrolyte to *in situ* generate the metal‐free *nido* carborane, [Bu_4_N]_2_[**^14C4^Cb**]. In a two‐compartment H‐cell separated by a fine glass frit, we added a solution of **^14C4^Cb** (0.01 M) in a 0.1 M [Bu_4_N][PF_6_] 1,2‐dimethoxyethane (DME) solution to the working compartment and a Ketjenblack slurry in a 0.1 M [Bu_4_N][PF_6_] DME solution to the counter compartment serving as a sacrificial reductant. GBE was performed until 2 mol equiv of electrons were passed *in situ*, generating the anion, **^14C4^Cb^2−^**, as the putative salt, [Bu_4_N]_2_[**^14C4^Cb**]. Conversion to **^14C4^Cb^2−^** was confirmed by the disappearance of the **^14C4^Cb** resonances and the emergence of 5 new resonances in the ^11^B{^1^H} NMR spectrum (Figure S13). A loss in theoretical yield is expected during GBE due to crossover across the glass frit in the H‐cell, adsorption onto the electrodes, and/or analyte degradation. Accordingly, we determined the exact concentration of **^14C4^Cb^2−^** by performing a back‐titration using a standardized solution of ferrocenium hexafluorophosphate, [Fc][PF_6_], which absorbs at 620 nm in the UV‐Vis region. One equiv of **^14C4^Cb^2−^** reacted with 2 equiv of Fc^+^ to regenerate **^14C4^Cb** and Fc, both of which do not absorb visible light at 620 nm. Correlating this to a calibration curve of the absorbance of [Fc][PF_6_] allowed us to determine the exact concentration of electrochemically‐generated **^14C4^Cb^2−^** (Figures S14‐S15).

We next turned to ^7^Li NMR titrations using the electro chemically‐generated **^14C4^Cb^2−^** solution and [Li(OEt_2_)_2.5_][TFAB] in DME as the Li^+^ source. The unlocked ^7^Li NMR spectra of these solutions are shown in Figure 4b. In the absence of any **^14C4^Cb^2−^**, we observed a sharp resonance corresponding to solvated Li^+^ at − 1.42 ppm (FWHM = 1.07 Hz). When < 1 equiv of **^14C4^Cb^2−^** was added relative to Li^+^, we observed an extremely broad ^7^Li resonance (**A**) between 0–2 ppm hidden in the baseline and a broadening resonance, **B**. With increasing equivalents of **^14C4^Cb^2−^**, resonance **B** shifted downfield from − 1.35 ppm (0.4 equiv, FWHM = 5.70 Hz) to − 0.8 ppm (0.9 equiv) with maximal broadening (FWHM = 16.33 Hz). Once > 1 equiv of **^14C4^Cb^2−^** was added, resonance **B** disappeared, favoring **A**. The very different chemical shift of **A** may suggest complete encapsulation of Li^+^ by the crown pocket, whereas resonance **B** (< 1 equiv of **^14C4^Cb^2−^**) may indicate rapidly interchanging crown‐encapsulated and outer‐sphere cage‐bound Li^+^ centers (Figure 3, box). Indeed, the complete disappearance of **B** upon addition of 1.1 equiv of **^14C4^Cb^2−^** suggests a 1:1 Li^+^:**^14C4^Cb^2−^** stoichiometry wherein the second, much weaker bound, outer‐sphere Li^+^ is likely replaced by the large excess of [Bu_4_N]^+^ cations from the supporting electrolyte. We attempted to perform variable temperature ^7^Li NMR experiments on these solutions; however, significant precipitation of dissolved species, likely the supporting electrolyte, impeded the acquisition of well‐defined spectra. While these ^7^Li NMR experiments qualitatively suggested strong Li^+^ binding by **^14C4^Cb^2−^**, the extreme broadness of the observed resonances precluded the determination of exact binding constants using this method.

### 2.2 Electrochemistry

**Figure 5:**
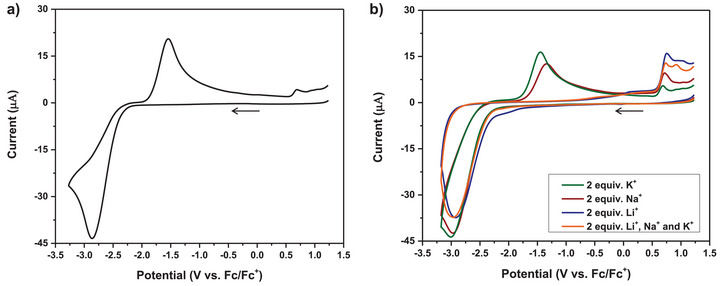
CVs of **^14C4^Cb** with and without added alkali metals to inform on the extent of metal binding to the complex. Conditions: 2.9 mM **^14C4^Cb** solution in DME with 0.1 M [Bu_4_N][PF_6_] supporting electrolyte, 50 mV/s scan rate, glassy carbon working electrode, Pt wire counter electrode, Ag_(s)_/AgOTf reference electrode. a) **^14C4^Cb** with no added metal b) **^14C4^Cb** with 2 equivalents of given alkali metals added as [Li(OEt_2_)_2.5_][TFAB] (Li^+^), [Na][PF_6_] (Na^+^), and [K][PF_6_] (K^+^) (5.8 mM each).

Electrochemistry is a powerful tool for probing binding interactions. For example, d‐block or f‐block metal complexes bearing crown moieties may experience anodic shifts in metal reduction potentials upon alkali metal complexation.^[^ ^39^, ^48^, ^49^, ^50^ ^]^ Similarly, redox‐active drugs have been shown to become redox‐inactive once bound to DNA.^[^ ^51^, ^52^ ^]^ The cyclic voltammogram (CV) of **^14C4^Cb** with alkali metal solutions offered an alternative method to measure **^14C4^Cb**–metal binding constants. In a 0.1 M [Bu_4_N][PF_6_] DME solution devoid of any metal species, we observed an irreversible 2e− redox event of **^14C4^Cb** with a formal reduction potential of *E*_1/2_ = −2.20 V versus the ferrocenium/ferrocene (Fc/Fc^+^) redox couple (Figure 5a). The large difference between the peak cathodic potential (*E*_p,c_ = −2.86 V) and the peak anodic potential (*E*_p,a_ = −1.54 V) suggested significant structural and solvation shell reorganization, which may be explained in part by C–C bond scission and cage rearrangement.

Subsequently, we acquired the CVs of solutions containing **^14C4^Cb** with 2 equivalents of alkali metals, Li^+^, Na^+^, and K^+^ added as [Li(OEt_2_)_2.5_][TFAB], [Na][PF_6_], and [K][PF_6_], respectively (Figure 5b). The TFAB salt of Li^+^ was selected for solubility reasons in preparing the stock solution given the limited solubility of [Li][PF_6_]. We note that, owing to the large excess of supporting [Bu_4_N][PF_6_] electrolyte, the [TFAB]− to [PF_6_]^−^ ratio is exceedingly low. The CV of **^14C4^Cb** with 2 equiv of [K][PF_6_] exhibited a shift in the peak oxidative potential by 70 mV (*E*_p,a_ = −1.47 V) accompanied by a 10% reduction in current compared to the CV without metal. In a solution with 2 equiv of Na^+^, a more substantial anodic potential shift of 200 mV (*E*_p,a_ = −1.34 V) and a greater decrease (31%) in peak current were observed. With 2 equiv of Li^+^, this trend was even more pronounced wherein the *nido→closo* oxidation event at − 1.54 V disappeared entirely, resulting in a 100% loss of current. We ascribe the loss of the oxidation current at this potential window to the direct binding of the alkali metal to our complex. The CV of **^14C4^Cb** with a mixed metal Li^+^/Na^+^/K^+^ solution (2 equiv each) closely resembled the CV with only Li^+^ present, lacking the *nido→closo* oxidation event.

In addition to the loss in return current observed upon metal addition, we observed a new oxidative event emerge at ∼ 0.7 V. While faintly observed in the absence of metal (Figure 5a), it is not present when scanning anodically first. In addition, we do not believe that it is a carborane‐ or metal‐based adsorption event. This was confirmed by testing whether the *nido* peak oxidation current (∼ −1.5 V) and the oxidation at ∼0.7 V scale proportionally with incremental cathodic sweeps with and without Li^+^ present. In a metal‐free solution of **^14C4^Cb**, we observed that the *nido* oxidation peak current increases as the CV is scanned more cathodically (Figure S31), consistent with the increased generation of the *nido* carborane. Additionally, we saw that intentionally scanning beyond the solvent window does not cause the *nido* oxidation to diminish, nor does the oxidation at ∼0.7 V increase dramatically, thus negating carborane‐based adsorption. With 0.87 mM (0.3 equiv) of Li^+^ in solution, we observed similar redox behavior where the *nido* oxidation increases with increasingly cathodic sweeps (Figure S30); however, here we see a marked increase in the oxidation event at ∼0.7 V when scanning cathodically. Scanning beyond the solvent window diminished the *nido* oxidation in favor of the ∼0.7 V oxidation event. Interestingly, when the CV scan is reversed at − 2.57 V (Figure S30, orange trace), no *nido* oxidation is observed, but the anodic event at ∼0.7 V is visible. Together, these data strongly suggest that the emerging oxidation at ∼0.7 V is due to metal‐bound *nido*‐carborane species reforming *closo*‐carborane (metal release). We further believe that the oxidation event at ∼0.7 V observed in the absence of added alkali ions is likely due to the presence of these trace metals in the supporting electrolyte solution.

We postulate that in situ‐generated *nido*‐carborane, **^14C4^Cb^2−^**, binds to alkali metal cations—generating the putative M_2_ **^14C4^Cb** (M = Li, Na, K) species—and becomes electrochemically deactivated to oxidation near − 1.54 V, instead undergoing oxidation at ∼ 0.7 V. Combining the metal–**^14C4^Cb^2−^** binding equilibrium and experimentally measured loss of the *nido*→*closo* peak current at varying metal concentrations then offers an approach to determining the binding constants of **^14C4^Cb^2−^** to the respective metals in solution. This was done using Equation 1, the derivation of which can be found in Section 5.3 of the Supporting Information:

(1) $$log\;\frac{i_{o}-\;i_{p,a}}{i_{p,a}}=log\;\frac{\Delta i}{i_{p,a}}=\;\;x\;log\left[M\right]+\log\;K$$

where *i*_o_ is the peak oxidation current in the absence of any metals, and *i*_p,a_ is the oxidation current once metal is added, and Δi is the magnitude of current decrease upon addition of Li^+^, Na^+^, or K^+^.^[^ ^52^, ^53^, ^54^, ^55^ ^]^ Plotting the logarithm of the relative current change $\log(\frac{\Delta i}{i_{p,a}})$ with respect to the concentration of metal ([M]), the y‐intercept would be equal to log *K*, while the number of metal ions binding to each **^14C4^Cb^2−^** would equal the slope (x). The obtained log *K* for each metal will be the summation of each individual alkali metal binding event to **^14C4^Cb^2−^** (e.g., log *K*_1_ + log *K*_2_).

**Figure 6:**
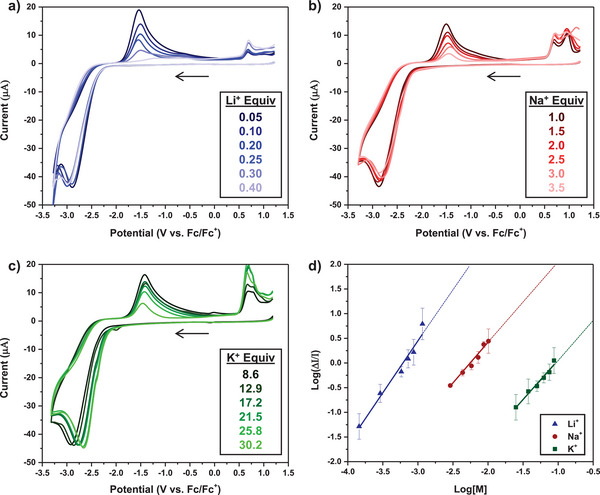
CV titration experiments of **^14C4^Cb** (2.9 mM) with varying equivalents of alkali metal to determine binding constants. All experiments were performed in 0.1 M [Bu_4_N][PF_6_] DME supporting electrolyte solutions at a 50 mV/s scan rate: a) [Li(OEt_2_)_2.5_][TFAB] CV titration; b) [Na][PF_6_] CV titration; c) [K][PF_6_] CV titration; d) linear regressions obtained from fitting in triplicate CV metal titrations to Equation 1. Data represent mean ± SD from three independent titrations.

We performed CV metal titrations in triplicate using a 2.9 mM solution of **^14C4^Cb** with varied equivalents of either [Li(OEt_2_)_2.5_][TFAB], [Na][PF_6_], or [K][PF_6_] in DME in 0.1 M [Bu_4_N][PF_6_] supporting electrolyte solutions (Figures 6, S19‐21).

Mixing Li^+^ with **^14C4^Cb** rapidly diminished the peak oxidative current of **^14C4^Cb^2−^** at −1.56 V (Figure 6a), becoming almost nonexistent once 0.40 equiv of Li^+^ was added. Fitting the CV data with Li^+^ to Equation 1 yielded a log *K* of 6.8 ± 0.6 with a stoichiometry of 2.1 ± 0.2 (Figure 6d, triangles). Similarly, mixing Na^+^ with **^14C4^Cb** also resulted in a decreased oxidation of **^14C4^Cb^2−^** (Figure 6b), albeit at much higher concentrations, requiring 3.5 equiv of Na^+^ to substantially suppress the oxidation. Fitting these data, we determined a log *K* of 3.7 ± 0.2 with 1.6 ± 0.1 Na^+^ ions binding to **^14C4^Cb^2−^** (Figure 6d, circles). Lastly, we observed much weaker binding with K^+^ (Figure 6c), and a prominent *nido*→*closo* oxidation peak still existed even with 30.2 equiv of K^+^. The CV data with K^+^ was fit to Equation 1, which resulted in a log *K* of 1.7 ± 0.2 and a stoichiometry of 1.6 ± 0.1 (Figure 6d, squares). From the binding constant measurements presented herein, **^14C4^Cb^2−^** appeared to bind Li^+^ cations with a substantially higher affinity compared to Na^+^ and K^+^, likely due to the smaller ionic radius of Li^+^.

We further benchmarked these experimental binding constants by the addition of competing complexing agents, commercial 12‐crown‐4 or 18‐crown‐6. A CV of **^14C4^Cb** with 0.3 equiv of Li^+^ and 0.7 equiv of 12‐crown‐4 (log *K*_Li_ = 2.14)^[^ ^15^ ^]^ revealed that the diminished oxidative current of **^14C4^Cb^2−^** did not recover (Figure S33), suggesting that **^14C4^Cb^2−^** outcompetes 12‐crown‐4 for Li^+^ complexation, consistent with our obtained log *K* value (6.8 ± 0.6). In contrast, a CV of **^14C4^Cb** with 30 equiv of K^+^ and 30 equiv of 18‐crown‐6 (log *K*_K_ = 4.21)^[^ ^56^ ^]^ revealed a restored oxidative current, confirming that 18‐crown‐6 outcompetes **^14C4^Cb^2−^** (log *K*_K_ = 1.7 ± 0.2) for K^+^ binding (Figure S34).

### 2.3 Electrochemical Simulations

**Figure 7:**
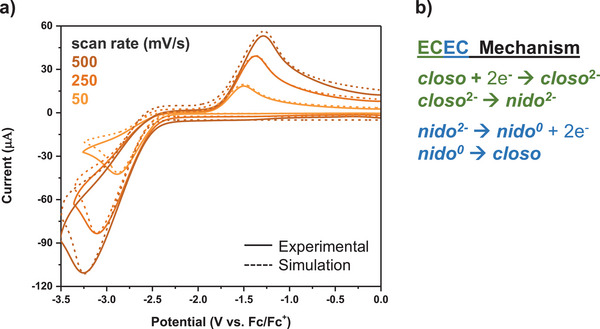
The carborane complex exhibits an ECEC mechanism. a) Simulated (dashed) and experimental CVs of 2.9 mM **^14C4^Cb** (solid) at 50 mV/s, 250 mV/s, and 500 mV/s scan rates. b) The corresponding ECEC mechanism for modeling the metal‐free CVs of **^14C4^Cb**.

Parallel to the analytical equilibrium binding model, we analyzed the voltammetry data from the **^14C4^Cb‐**alkali metal solutions by simulating these CVs and determining the log *K* values independent of Equation 1. DigiSim version 3.03b was used to simulate the voltammetry experiments.^[^ ^57^ ^]^ We first simulated the CVs of **^14C4^Cb** in the absence of alkali metals at variable scan rates to converge to the electrochemical parameters of the **^14C4^Cb**/**^14C4^Cb^2−^** redox couple (Figure 7a). Interestingly, we found the very broad reduction and oxidation events of **^14C4^Cb** are best modeled by an ECEC mechanism. In this mechanism, the neutral *closo* species is reduced to a *closo^2−^* dianion that undergoes rapid C–C bond scission to generate the *nido* ^2−^ dianion. During the reverse sweep, the *nido* ^2−^ dianion is oxidized to a short‐lived neutral *nido^0^* species that rapidly reforms the cage C–C bond to revert back to the *closo* species (Figure 7b).

**Figure 8:**
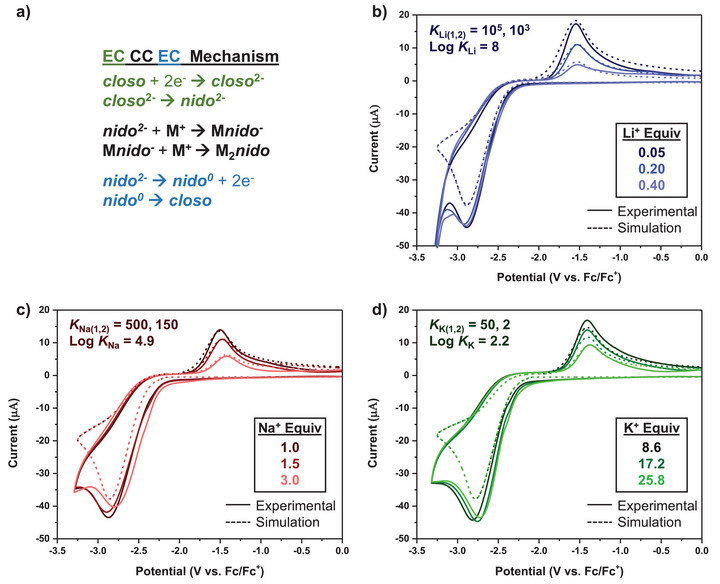
a) The proposed ECCCEC mechanism for modeling CVs of **^14C4^Cb^2−^** binding to two alkali metal (M^+^) cations. b) Simulated (dashed) and experimental (solid) CVs of **^14C4^Cb** with 0.05, 0.20, and 0.40 equiv of Li^+^. c) Simulated (dashed) and experimental (solid) CVs of **^14C4^Cb** with 1.0, 1.5, and 3.0 equiv of Na^+^. d) Simulated (dashed) and experimental (solid) CVs of **^14C4^Cb** with 8.6, 17.2, and 25.8 equiv of K^+^.

We next determined the fitting parameters—the heterogenous electron transfer rates (*k*_o_), charge transfer coefficients (*α*), diffusion coefficients (*D*), etc.—and input these parameters for further CV simulations, now in the presence of varying equivalents of alkali metals (see Supporting Information Section S6). Simulations suggest that **^14C4^Cb^2−^** undergoes stepwise complexation reactions with alkali metals, adding two chemical steps to the ECEC mechanism to create an ECCCEC mechanism (Figure 8a). We entered the bulk solution concentrations of Li^+^, Na^+^, or K^+^ used experimentally, alongside diffusion coefficients for each cation reported from literature.^[^ ^58^, ^59^, ^60^ ^]^ Accordingly, the equilibrium of the forward versus backwards chemical reaction (*k*_f_/*k*_b_) was optimized, and the binding constant (*K*) for each metal was obtained by fitting simulated voltammetry to experimental voltammetry (Figure 8b‐d).

From these CV simulations, we determined log *K* values of **^14C4^Cb^2−^** for Li^+^ (log *K*_Li_ = 8), Na^+^ (log *K*_Na_ = 4.9), and K^+^ (log *K*_K_ = 2.2) (Figure 8b‐d). The simulations model two binding events (*K_1_* and *K_2_*; the **CC** in EC**CC**EC); thus, the overall log *K* is the summation of both **CC**. The simulated values follow the same trend as the analytical binding model with a binding preference for Li^+^ over Na^+^ and K^+^. Additionally, the simulated log *K* values per metal cation match well with experimental values (Table 1).

**Table 1:** Log *K* binding values of **^14C4^Cb** with Li^+^, Na^+^, or K^+^ determined through experimental and simulated methods.

Alkali Metal	Experimental Method			Simulated Method	
M^+^	Total Log *K*	Stoichiometry	Log *K* per M^+^	Total Log *K*	Log *K* per M^+^
Li^+^	6.8 ± 0.6	2.1 ± 0.2	3.4^[^ ^a^ ^]^	8.0	4.0
Na^+^	3.7 ± 0.2	1.6 ± 0.1	2.4^[^ ^b^ ^]^	4.9	2.45
K^+^	1.7 ± 0.2	1.6 ± 0.1	1.1^[^ ^b^ ^]^	2.2	1.1

^[a]^Calculated assuming metal binding stoichiometry rounded to 2.

^[b]^Calculated assuming metal binding stoichiometry rounded to 1.5.

Looking into the concentration profiles of each chemical species as a function of time and position using DigiSim at this juncture informed us about the limitations of the equilibrium binding model described earlier (Equation 1). Under the simulated model, the electrogenerated **^14C4^Cb^2−^** rapidly binds to Li^+^, depleting nearly all the free Li^+^ proximate to the electrode surface under the bulk solution Li^+^ concentrations used (∼ 0.4 equiv). This led to significant mass transport of free Li^+^ to the electrode, where it was coordinated by the **^14C4^Cb^2−^** generated at the electrode. This concentration gradient explained several observations with the CVs of the **^14C4^Cb**/Li^+^ system. The *nido*→*closo* oxidative current is entirely suppressed by the time just 0.5 equiv of bulk Li^+^ is present in solution; this is possible because additional free Li^+^ away from the electrode rapidly diffuses toward the electrode. In simulations where we hypothetically assumed that the Li^+^ diffusion coefficient was more rapid by a factor of 10, we found merely 0.25 equiv of Li^+^ would eliminate the *nido*→*closo* oxidation wave (Figure S43). Thus, the Li^+^ to **^14C4^Cb^2−^** binding constant is too large to compute accurately, and the value obtained here *represents a lower‐limit value* for log *K*_Li_ because the diffusion of Li^+^ ultimately dictates the relative loss of the *nido*→*closo* oxidative current, rather than the precise value of the **^14C4^Cb**/Li^+^ binding constant.

For the cases of Na^+^ and K^+^, the concentrations of the alkali metals were significantly greater than the concentrations of the **^14C4^Cb^2−^**; hence, the simulations show negligible changes in the concentration of these cations, and the loss of the *nido*→*closo* oxidation current is reflective of the values of the binding constant to these metals. At faster scan rates of 1 V/s and 2 V/s, simulations of **^14C4^Cb** with Li^+^ demonstrated that Li^+^ is no longer fully depleted near the electrode and does not hinder the chemical reaction with **^14C4^Cb^2−^**. We paired these simulations with experimental voltammetry and obtained a log *K*_Li_ of 8, which is consistent with the binding constant obtained at a 50 mV/s scan rate (Figures S41, S42).

### 2.4 Limitations to Methods

We have derived a binding model (Equation 1) for redox‐active complexing agents undergoing multiple consecutive electrochemical and chemical reactions. The experimental method, performed in triplicate, suggests an error of approximately 10% of the true value. The accuracy of this does not compete with traditional ^1^H NMR binding methods. However, it allows for obtaining log *K* values that would be otherwise unattainable due to synthetic challenges and complexities of multiple coordination environments. The experimental binding model does not account for mass transport of the cations, an integral component on the timescale of a CV. We suggest that the experimental method be paired with simulated CVs for obtaining a modeled concentration gradient to verify that the reagent is not fully depleted at the electrode surface. Furthermore, we advise performing the CV titration experiments in triplicate because the peak oxidation currents obtained herein vary with each scan. The simulated voltammetry method using DigiSim 3.0b is limited by stepwise chemical reactions, which increases the complexity of the modeled system.

## 3 Conclusions

In this report, we have described the synthesis and electrochemical behavior of a fundamentally new class of highly selective carborane‐crown Li‐capture agent, **^14C4^Cb**. Weak, negligible binding to Li^+^ was observed in the neutral *closo* state, whereas strong binding was observed in the cage‐opened reduced *nido* state, **^14C4^Cb^2−^**. Structural studies on the related **K_2_^14C4^Cb** revealed a crown‐captured K^+^ bound by two of the four crown oxygen atoms, as well as through cation‐π interactions with the carborane. A second K^+^ is bound outer‐sphere through B–H agostic interactions, forming a polymeric structure. DFT studies on the **Li_2_^14C4^Cb** congener revealed a similar structural arrangement. Attempts to obtain binding constants of **^14C4^Cb^2−^** to Li^+^, Na^+^, and K^+^ ions by NMR spectroscopy in acetonitrile were unsuccessful and yielded only qualitative indications of strong **^14C4^Cb^2−^**/Li^+^ binding. Using instead cyclic voltammetry, we derived a new binding model (Equation 1) for the complexation of the **^14C4^Cb**/**^14C4^Cb^2−^** couple undergoing ECEC (in the absence of alkali metals) or ECCCEC (with alkali metals) mechanisms through observed experimental phenomena paired with simulated voltammetry. The following log *K*_metal_ (experimental; *simulation*) values were obtained: log *K*_Li_ (6.8 ± 0.6; *8.0*), log *K*_Na_ (3.7 ± 0.2; *4.9*), log *K*_K_ (1.7 ± 0.2; *2.2*). Simulated voltammetry revealed that rapid mass transport of Li^+^ limited our ability to obtain binding constants via experimental CV, which neglected diffusion toward the electrode surface in the calculation. In essence, the Li^+^ to **^14C4^Cb^2−^** binding constant is too large to compute accurately, and the higher obtained simulated value (log *K*_Li_ = 8.0) *represents a lower‐limit value* for log *K*_Li_. We attribute the strong binding of Li^+^ over Na^+^ and K^+^ to both the favorable redox‐tunable crown cavity size of the **^14C4^Cb/^14C4^Cb^2−^** couple, combined with strong coulombic interactions in the reduced *nido* state. In summary, while this platform is not currently suitable for DLE applications due to its highly reducing nature, we believe that the crown‐carborane platform itself may eventually offer a new, rapid, and highly selective technique for Li^+^ capture in electrochemical DLE technologies. Indeed, we are currently focusing on shifting the redox potentials of such crown platforms to make them suitable for biphasic or heterogeneous Li^+^ capture/release from aqueous streams and are focusing on more reversible platforms for real‐world DLE applications.

## Supporting Information

Experimental procedures and characterization data are available in the Supporting Information. The authors have cited an additional reference within the Supporting Information.^[^ ^31^, ^36^, ^37^, ^42^, ^43^, ^44^, ^45^, ^46^, ^47^, ^52^, ^53^, ^54^, ^55^, ^58^, ^59^, ^60^, ^61^ ^]^

Deposition Number(s) 2338535 (for **^14C4^Cb**) and 2338536 (for **K_2_^14C4^Cb**) contain(s) the supplementary crystallographic data for this paper. These data are provided free of charge by the joint Cambridge Crystallographic Data Centre and Fachinformationszentrum Karlsruhe Access Structures service.

## Conflict of Interest

The authors declare that a patent application related to this work has been filed (PCT/US2023/01 9388). G. Ménard, L. Sepunaru, Z. Wang, and S. Heinrich are listed as inventors. The authors declare no other competing interests.

## Supporting information



Supporting Information

## Data Availability

The data that support the findings of this study are available in the supplementary material of this article.
