# Supplementary material for: A Redox‐Tunable Carborane Crown: Toward Highly Selective Electrochemical Lithium Capture
Source: Chemistry. 2025 Nov 17;31(72):e02902. doi: 10.1002/chem.202502902 (PMC12731541; doi:10.1002/chem.202502902)
Supplement: Supplementary file 1 — Supporting Information [file CHEM-31-e02902-s001.pdf]

Electronic Supporting Information for:

A Redox-Tunable Carborane Crown: Towards Highly Selective Electrochemical Lithium Capture

Shannon Heinrich,[1] Zongheng Wang,[1] Jitendrasingh Rajpurohit,[3] Ashley Yeow,[1] Roman Dobrovetsky,[2] Lior Sepunaru,[1] and Gabriel Ménard*[1,3]

[1] Department of Chemistry and Biochemistry, University of California, Santa Barbara, California 93106, United States.

[2]School of Chemistry, Raymond and Beverly Sackler Faculty of Exact Sciences, Tel Aviv University, Tel Aviv 69978, Israel.

[3]Department of Chemistry, University of Calgary, 2500 University Drive NW, Calgary, Alberta T2N 1N4, Canada.

Table of Contents

S1. Materials and Physical Measurements 2
S2. Synthesis of Compounds 3
S3. *Closo* NMR Binding 11
S4. *Nido* NMR Binding 11
S4.1 *Galvanostatic Bulk Electrolysis* 11
S4.2 *UV-Visible Calibration Curve* 13
S4.3 *Nido NMR Binding Procedure* 14
S5. Electrochemistry 15
S5.1 *Randles-Ševčík Analysis* 15
S5.2 *CV Titration Procedure* 17
S5.3 *CVs of* ***^{14C4}Cb*** *with Li^+, Na^+, or K^+* 18
S5.3 *Binding constant equation* 19
S5.4 *CV titration linear regressions* 20
S5.5 *CV Controls* 22
S6. Simulated Voltammetry 28
S6.1 *Simulation General Parameters* 28
S6.2 *CV Simulations* 29
S6.3 *CV Simulation Controls* 32
S7. Supplemental Figure 34
S8. DFT Studies 34
S9. References 43

S1. Materials and Physical Measurements

General Considerations

All manipulations were performed under an atmosphere of dry, oxygen-free N_2 or Ar by means of standard Schlenk or glovebox techniques (MBraun equipped with a -38 °C freezer, or VAC gloveboxes). Molecular sieves (3Å, Fisher) were activated by heating (~200 °C) under a dynamic vacuum (<40 mtorr) overnight and brought into the glovebox. Hexanes, pentane, DCM, diethyl ether, THF and benzene were dried on an MBraun solvent purification system. Glyme, DMF, and acetonitrile were dried over CaH_2 for several days prior to distillation. All solvents were stored over molecular sieves (3Å, Fisher). All deionized water was obtained from a MilliQ purifier with a resistivity of 18.2 MΩ.cm.

Ortho-carborane was purchased from Boron Specialties and sublimed before use. Potassium hydride was purchased from Sigma Aldrich and washed with hexanes prior to use. Tetrabutylammonium hexafluorophosphate was purchased from Oakwood Chemicals and purified twice by dissolving in hot ethanol and precipitating out with deionized water and washed with additional deionized water then dried at 100 ºC under vacuum for 24 h. Lithium hexafluorophosphate was purchased from TCI Chemicals and recrystallized before use. Potassium hexafluorophosphate was purchased from Matrix Scientific and recrystallized prior to use. Sodium hexafluorophosphate was purchased from Acros Organics and used as received. Lithium tetrakis(pentafluorophenyl)borate ethyl etherate ($[Li(OEt_2)_{2.5}][TFAB]$) was purchased from Sigma Aldrich and used as received. 1,2-diol-*o*-carborane[1] (1,2-$(OH)_2$-Cb) and the tosyl linker, 2,3-bis(3-[p-toluenesulfonyloxy]propoxy)-2,3-dimethylbutane,[2] were prepared using literature procedures. Ketjenblack EC600-JD was purchased from MSE Supplies. The Ketjenblack was dried for 48 h in a 175 °C oven and ground in a glass mortar and pestle under inert atmosphere prior to use.[3]

Physical Measurements

NMR spectra were obtained on Bruker Avance NEO 500 MHz spectrometer and referenced to residual solvent resonances of chloroform ($CDCl_3$) or tetrahydrofuran (C_4D_8O) or externally (^{11}B: 85% $(Et_2O)BF_3$, ^{7}Li: 9.7 M $LiCl/D_2O$). Chemical shifts (δ) are recorded in ppm.

Elemental analyses (C, N, H) were recorded at the University of California, Santa Barbara using an Exeter Analytical CE440 elemental analyser for air stable compounds. Elemental analyses (C, N, H) for air-sensitive compounds were recorded at University of California, Berkeley using a ThermoFisher Flash Smart Elemental analyzer.

X-ray crystallography data were collected at the University of California, Santa Barbara on a Bruker KAPPA APEX II diffractometer equipped with an APEX II CCD detector using a TRIUMPH

monochromator with a Mo Kα X-ray source (wavelength α = 0.71073 Å). The crystals were mounted on a cryoloop under Paratone-N oil, and all data were collected at 100(2) K using an Oxford nitrogen-gas cryostream system. A hemisphere of data was collected using ω scans with 0.5° frame widths (ω, angle between the X-ray source and the sample). Data collection and cell-parameter determination were conducted using the SMART program. Integration of the data frames and refinement of the final cell parameters were performed using SAINT software. Absorption correction of the data was carried out using SADABS. Structure determination was done using direct or Patterson methods and difference Fourier techniques. All hydrogen atom positions were idealized and rode on the atom of attachment. Structure solution, refinement, graphics, and creation of publication materials were performed using SHELXTL or $OLEX^2$.

Cyclic voltammetry was performed using a CH Instruments Electrochemical Analysis potentiostat equipped with a 3-mm-diameter glassy carbon working electrode, 5 mM Ag/AgOTf reference electrode (BASi, non-aqueous reference electrode kit) and a Pt-wire counter electrode using a $[Bu_4N][PF_6]$ (0.1 M) solution as the supporting electrolyte. Cyclic voltammograms were referenced to the Fc/Fc^+ redox couple. The glassy carbon working electrode was polished between each scan with diamond slurry (3 μm, 1 μm, 0.25 μm and 0.05 μm, Buehler MetaDiTM Supreme) on polishing pads (Buehler MasterTex) for 2 minutes per pad and sonicated in DI water for ~ 5 s. The Pt-wire counter electrode was rinsed with acetone and heated until bright red with a propane flame.

Galvanostatic Bulk Electrolysis cycling experiments were carried out using a Biologic VMP3 potentiostat/galvanostat and carried out inside a nitrogen glovebox. The full experimental setup for electrolysis experiments is described in section S4.

UV-Visible Spectroscopy was performed using an Agilent 8543 UV-Visible Spectrophotometer with screw top quartz cuvettes supplied from Starna Cells Inc.

S2. Synthesis of Compounds

1,2-((6,6,7,7-Me$_4$)14-crown-4)-*o*-carborane (^{14C4}Cb)

Under an inert atmosphere, 1,2-$(OH)_2$-Cb (0.360 g, 2.04 mmol, 1 equiv.) was dissolved in 5 mL THF and cooled to -25 °C. To this, a suspension of KH (0.168 g, 4.2 mmol, 2.05 equiv.) in THF was added and the resulting pale-yellow solution was stirred for 0.5 h at r.t. All volatiles were removed under reduced pressure and the reaction mixture was redissolved in minimal DMF. To this, 2,3-bis(3-[*p*-toluenesulfonyloxy]propoxy)-2,3-dimethylbutane (1.11 g, 2.04 mmol, 1 equiv.) in 10 mL of DMF was added dropwise. The solution was stirred overnight at 80 ºC. The volatiles were then removed under reduced pressure. The residue was redissolved in DCM and washed with water and brine. The organic layer was

then separated, dried over anhydrous $MgSO_4$, and filtered. The solvent was removed, and the residue was purified by column chromatography (5% ethyl acetate / 95% hexanes). Yield: (0.250 g, 32.8%) Single crystals suitable for XRD studies were obtained by recrystallization from a saturated diethyl ether solution at −25 °C. **^{1}H NMR (500 MHz, $CDCl_3$)** δ 3.96 (t, *J* = 5.4 Hz, 4H), 3.54 (t, *J* = 6.3 Hz, 4H), 1.83 (p, *J* = 6.1 Hz, 4H), 1.14 (s, 12H). **^{11}B{^{1}H} NMR (160 MHz, $CDCl_3$)** δ -13.08, -13.95, -15.10, -17.80. **^{13}C NMR (126 MHz, $CDCl_3$)** δ 109.3, 80.6, 73.0, 58.1, 30.6, 21.5. *Anal. Calcd.* for $B_{10}C_{14}O_4H_{34}$: C, 44.90; H, 9.15. *Found*: C, 45.27; H, 9.26.

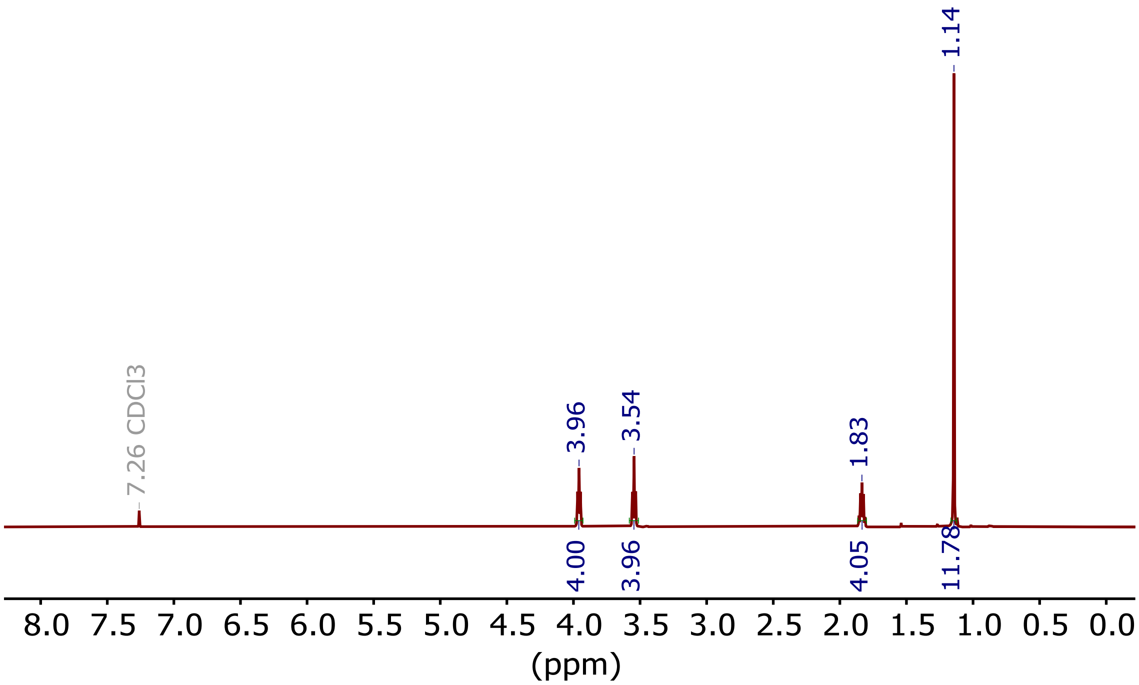

Figure S1. 500 MHz ^{1}H NMR of **^{14C4}Cb** in $CDCl_3$

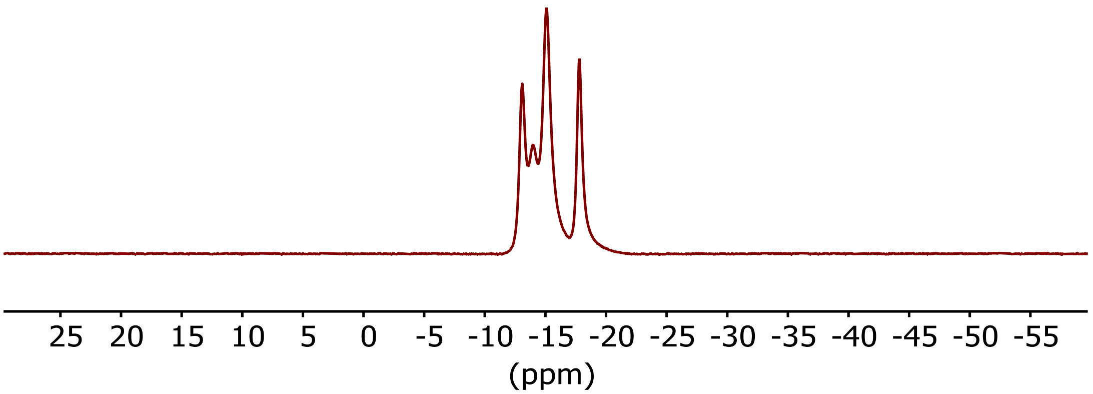

Figure S2. 160 MHz $^{11}B\{^1H\}$ NMR of **^{14C4}Cb** in $CDCl_3$.

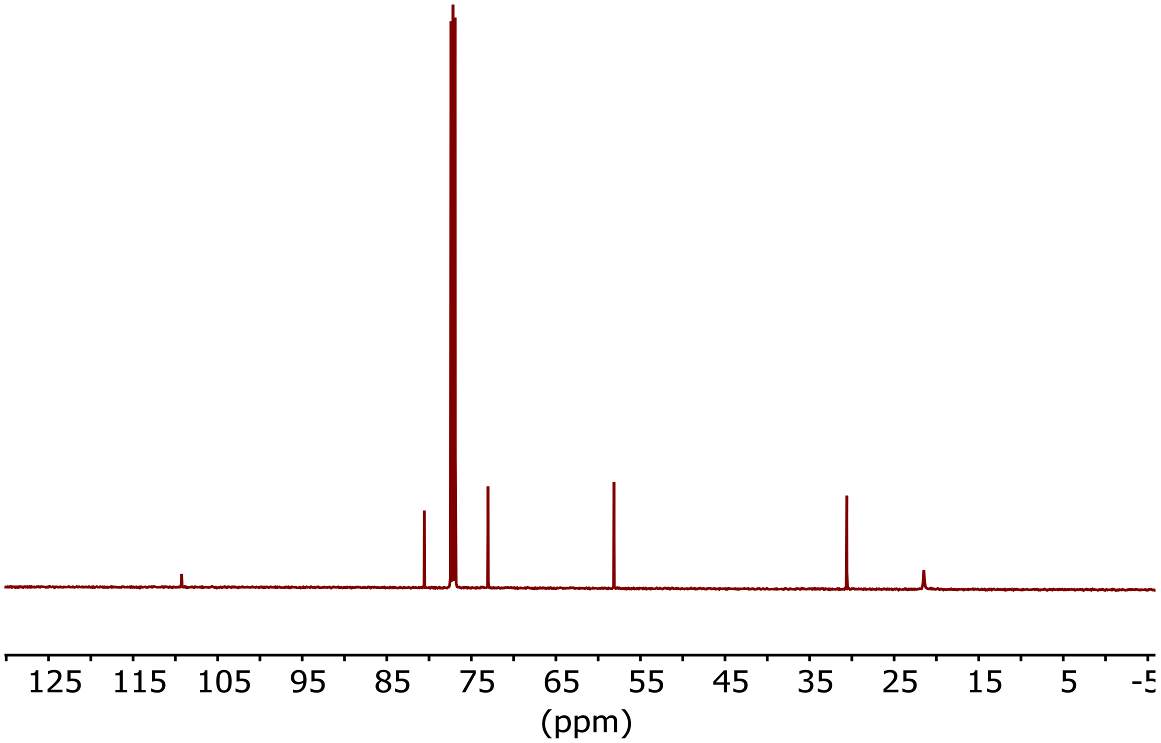

Figure S3. 126 MHz $^{13}C\{^1H\}$ NMR of **^{14C4}Cb** in $CDCl_3$.

K_2[1,2-((6,6,7,7-Me$_4$)14-crown-4)-*o*-carborane] (K_2^{14C4}Cb)

Naphthalene (0.016 g, 0.12 mmol, 2 equiv.) was dissolved in dry THF and cooled to -78 ºC. Excess solid K was added and the reaction stirred at r.t. for 0.5 h. The deep green solution was filtered over a celite plug and extracted with additional THF. **^{14C4}Cb** (0.024 g, 0.06 mmol, 1 equiv.) was dissolved in THF and cooled to -78 ºC and the potassium naphthalenide solution was added dropwise. The reaction stirred for an hour at r.t. The volatiles were removed *in vacuo*. Then, the reaction was stirred with diethyl ether (5 mL × 3) and decanted. The reaction was dissolved in acetonitrile and filtered through a celite plug. The solution was saturated then recrystallized at -25 ºC producing colorless crystals suitable for XRD studies. Yield: (0.023 g, 84.9%). **^{1}H NMR (500 MHz, CD_3CN)** δ 3.96 (t, *J* = 7.6 Hz, 4H), 3.42 (t, *J* = 5.6 Hz, 4H), 1.75 (p, *J* = 6.8 Hz, 4H), 1.10 (s, 12H). **^{11}B{^{1}H} NMR (160 MHz, CD_3CN)** δ -8.06, -23.59. **^{13}C NMR (126 MHz, CD_3CN)** δ 81.4, 70.1, 68.2, 59.0, 31.4, 20.4. *Anal. Calcd.* For $K_2B_{10}C_{14}O_4H_{34}$: C, 37.14; H, 7.57. *Found*: C, 37.09; H, 7.28.

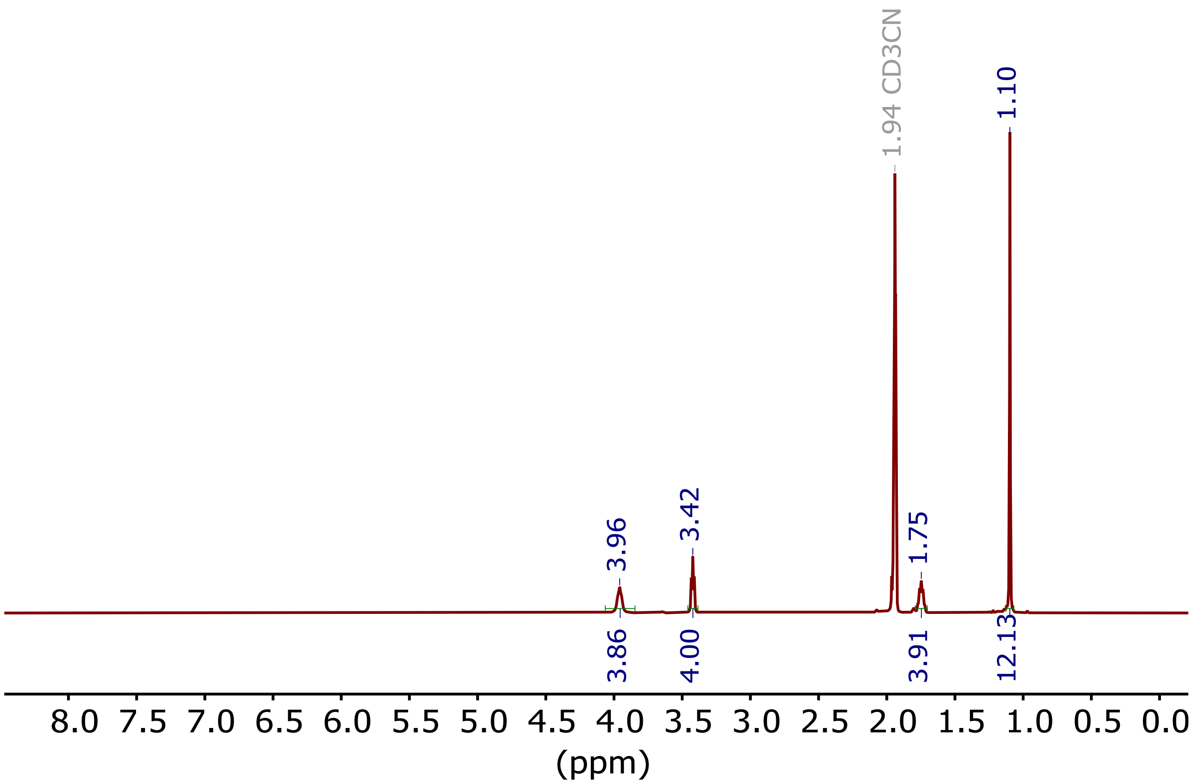

Figure S4. 500 MHz ^{1}H NMR of **K_2^{14C4}Cb** in CD_3CN.

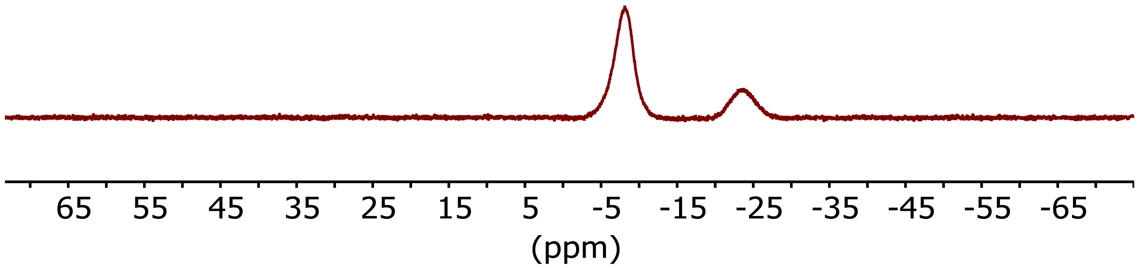

Figure S5. 160 MHz $^{11}B\{^1H\}$ NMR of **K_2^{14C4}Cb** in CD_3CN.

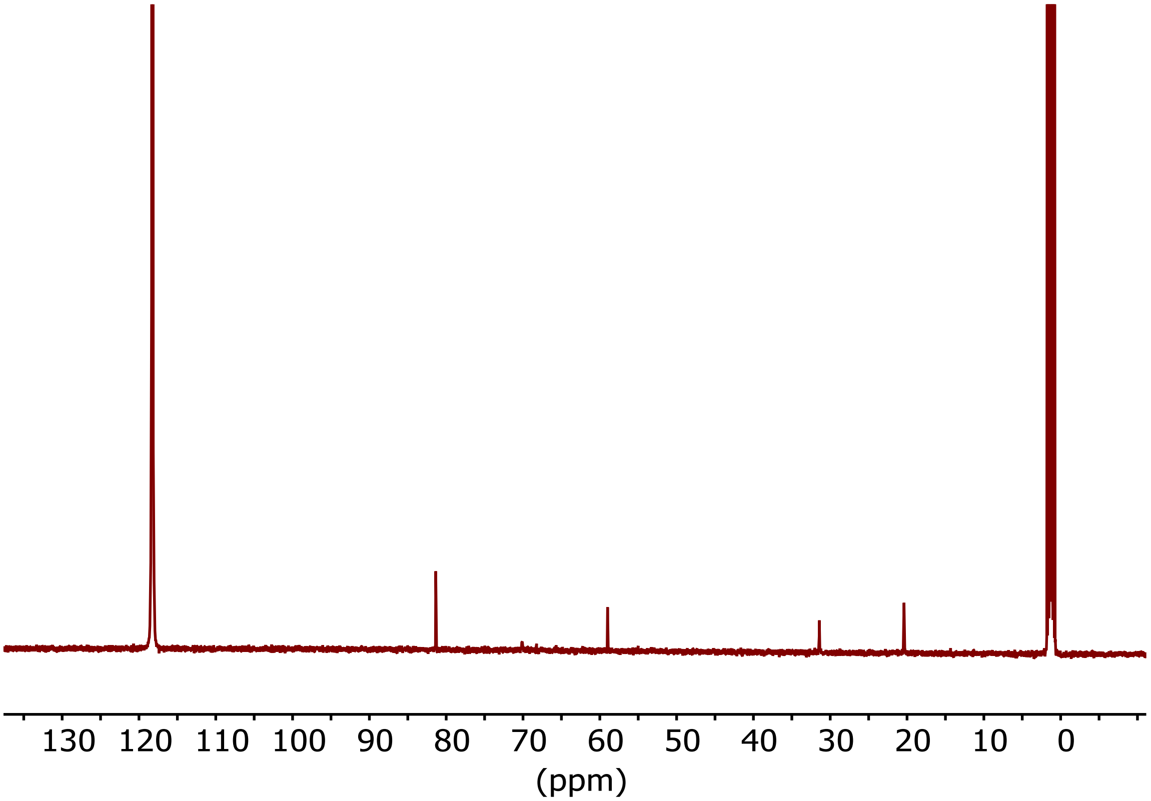

Figure S6. 126 MHz $^{13}C\{^1H\}$ NMR of **K_2^{14C4}Cb** in CD_3CN.

Li_2[1,2-((6,6,7,7-Me_4)14-crown-4)-*o*-carborane] (**Li_2^{14C4}Cb**)

K_2^{14C4}Cb (0.0215 g, 0.046 mmol) was dissolved in 1 mL of acetonitrile. [Li(OEt_2)$_{2.5}$][TFAB] (0.0829 g, 0.095 mmol) was dissolved in 1 mL of acetonitrile. It was added to the stirring carborane and stirred for 48 hours. Afterwards, the stirring was discontinued. The reaction was filtered through a celite plug and then taken to dryness, yielding a white, powdery mixture of **Li_2^{14C4}Cb** and K(OEt_2)TFAB. **^{1}H NMR (500 MHz, CD_3CN)** δ 3.85 (t, *J* = 6.1 Hz, 2H), 3.61 (t, *J* = 5.9 Hz, 2H), 3.54 (t, *J* = 5.6 Hz, 2H), 3.40 (hidden under Et_2O, 2H), 1.72 (p, *J* = 6.0 Hz, 2H), 1.68 (p, *J* = 5.8 Hz, 2H), 1.15 (s, 6H), 1.13 (s, 6H). **^{11}B{^{1}H} NMR (160 MHz, CD_3CN)** δ -5.91, -12.80, -13.45, -14.03, -15.80. **^{7}Li NMR (194 MHz, CD_3CN)** δ -2.96. **MALDI-HRMS** for $C_{14}H_{35}B_{10}LiO_4$ ([M–Li^++H^+]$^+$): expected 382.3698; observed 382.3630.

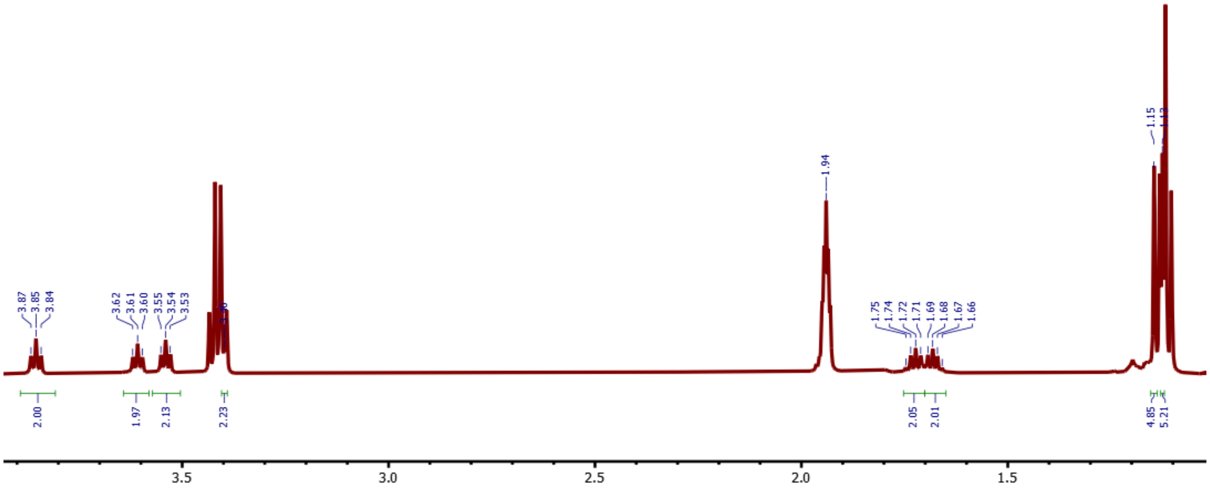

Figure S7. 500 MHz ^{1}H NMR of **Li_2^{14C4}Cb** in CD_3CN (1.94 ppm) with residual diethyl ether (3.41 and 1.12 ppm).

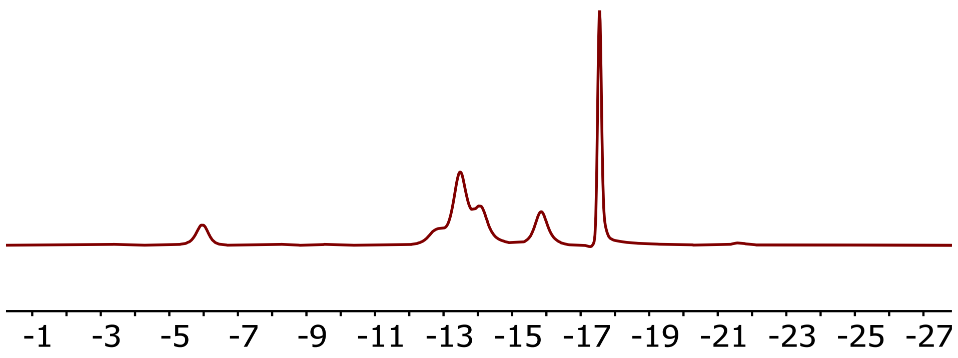

Figure S8. 160 MHz $^{11}B\{^{1}H\}$ NMR of **Li_2^{14C4}Cb** in CD_3CN with residual [K][TFAB] (-17.5 ppm).

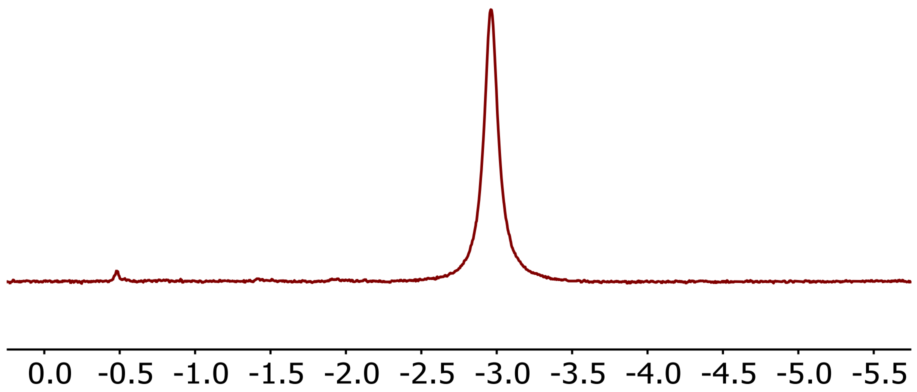

Figure S9. 194 MHz ^{7}Li NMR of **Li_2^{14C4}Cb** in CD_3CN.

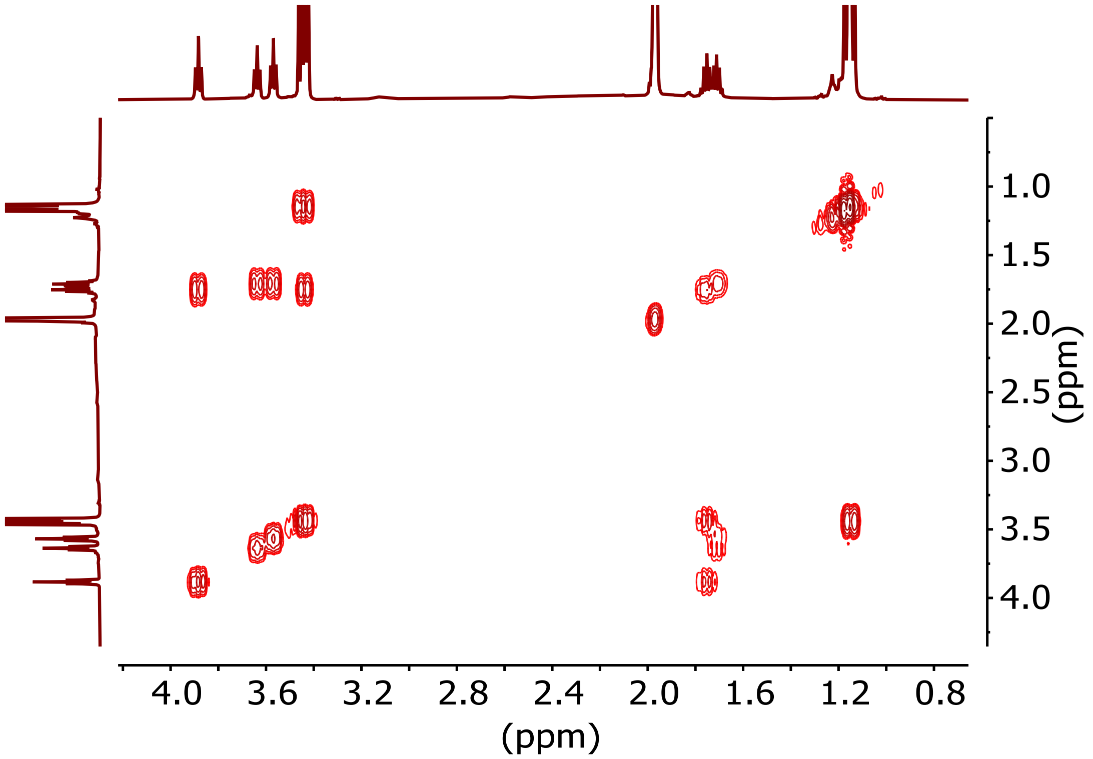

Figure S10. 2D NMR ^{1}H–^{1}H COSY (500 MHz) spectra of **Li_2^{14C4}Cb** in CD_3CN.

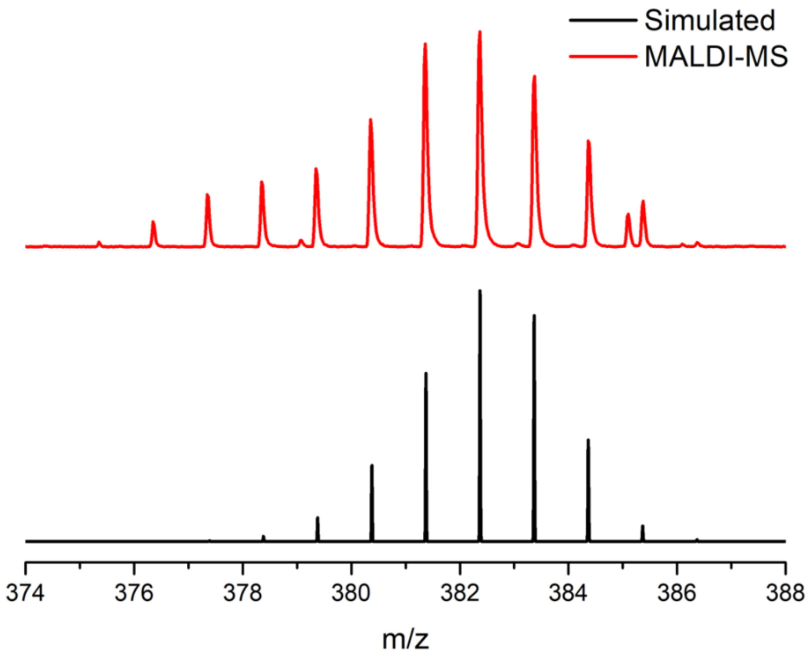

Figure S11. Simulated and experimental MALDI-HRMS of **Li_2^{14C4}Cb** ([M]) corresponding to the [M–Li^++H^+]$^+$ ion peak (expected 382.3698, observed 382.3630).

S3. *Closo* NMR Binding

Stock solutions of 20 mM **^{14C4}Cb** and 60 mM of lithium hexafluorophosphate ($LiPF_6$) in deuterated THF were prepared. To a glass vial, the desired amount of each stock solution was dispensed using a Hamilton syringe. The sample was then diluted to a final volume of 0.50 mL with deuterated THF. The contents of the vial were thoroughly mixed and transferred to an NMR tube. ^1H and ^7Li NMR spectra were collected on a Bruker Avance NEO 500 MHz. ^7Li NMR spectra were collected (128 scans) with 1 second T_1 relaxation delay. The ^1H and ^7Li spectra were referenced to the residual solvent d-THF peak.

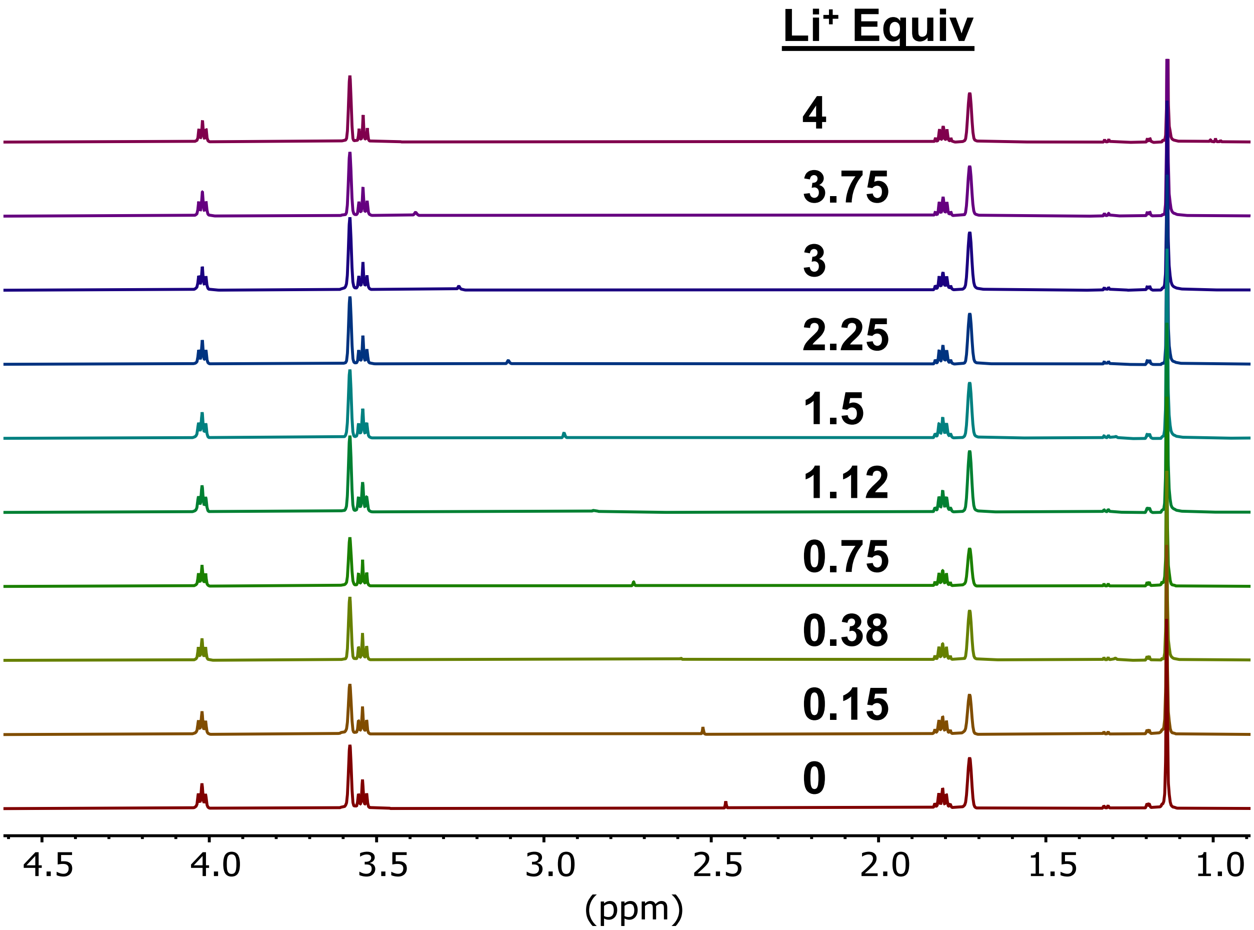

Figure S12. 500 MHz ^1H NMR spectra of **^{14C4}Cb** in THF-d_8 with varied equivalents of $LiPF_6$.

S4. *Nido* NMR Binding

S4.1 *Galvanostatic Bulk Electrolysis*

Galvanostatic Bulk Electrolysis (GBE) experiments were carried out using a Biologic VMP3 potentiostat/galvanostat under inert atmosphere. Two-electrode GBE was performed in a nitrogen

glovebox utilizing a two compartment H-cell with a glass frit separator, a stir bar in each compartment, and reticulated vitreous carbon (RVC) foam electrodes for both the working and counter electrodes. The RVC foam electrodes consisted of a ~5 cm steel rod inserted into 100 PPI Duocel® RVC foam core (length ~3 cm; diameter ~3mm), with a tap bore (length ~5 mm; diameter ~2 mm), which was filled with molten gallium to fuse the steel connector to the RVC foam. Each electrode has an end-to-tip resistance of <30 Ω. The RVC electrodes were rinsed with acetone and dried.

Reduction (Charging): The counter compartment consisted of 300 mg of Ketjenblack suspended in 8 mL of a 0.1 M solution of $[Bu_4N][PF_6]$ in DME. The working compartment consisted of ^{14C4}**Cb** (0.063 mmol, 1.0 equiv) dissolved in 6 mL of a 0.1 M solution of $[Bu_4N][PF_6]$ in DME. A charging current of -140 µA with a -12 C charge cutoff was utilized, resulting in a ca. 100% SOC after 24 h assuming 100% columbic efficiency. Upon completion, the working compartment solution was monitored by $^{11}B\{^1H\}$ NMR spectroscopy to reveal full conversion to $[Bu_4N]_2$[^{14C4}**Cb**] (Figure S12).

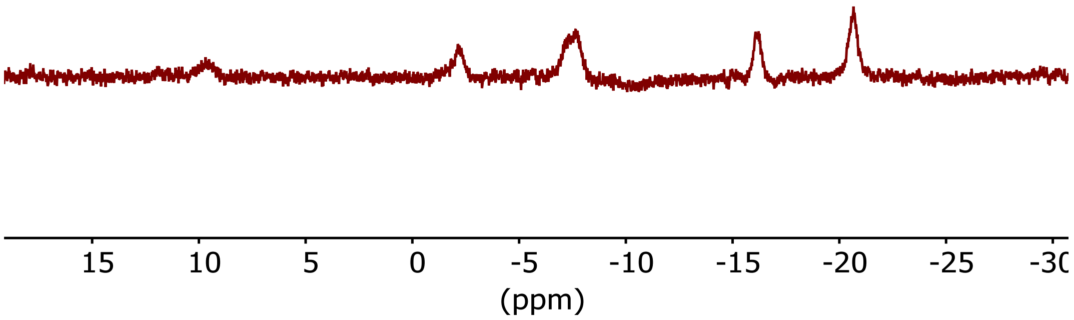

Figure S13: Unlocked 160 MHz $^{11}B\{^1H\}$ NMR spectra of electrochemically generated ^{14C4}**Cb**$^{2-}$ in DME with 0.1 M $[Bu_4N][PF_6]$ supporting electrolyte.

S4.2 *UV-Visible Calibration Curve*

A 3 mM stock solution of ferrocenium hexafluorophosphate ($FcPF_6$) in 0.1 M $[Bu_4N][PF_6]$ in DME was prepared. Stepwise dilutions were prepared by mass using the density of the solution 0.1 M $[Bu_4N][PF_6]$ in DME, 0.8816 g/mL. UV-Vis spectra were recorded for 5 different concentrations of $FcPF_6$ with absorbance at 620 nm ranging between 0.1–1 for linearity, adhering to Beer-Lambert's law (Figure S13). A calibration curve was constructed plotting absorbance at 620 nm versus concentration of $FcPF_6$ and a linear regression analysis was employed (Figure S14). An aliquot of the working compartment solution was then added to a known volume and concentration of ferrocenium hexafluorophosphate in 0.1 M $[Bu_4N][PF_6]$/DME. The absorbance of the solution was measured by UV-Vis spectroscopy. The concentration of $FcPF_6$ remaining was found from the calibration curve and the amount of $FcPF_6$ that reacted with ^{14C4}**Cb**$^{2-}$ was used to determine the concentration of ^{14C4}**Cb**$^{2-}$ in the working compartment solution (Figure S14). The remaining ^{14C4}**Cb**$^{2-}$ solution was utilized for subsequent NMR binding studies.

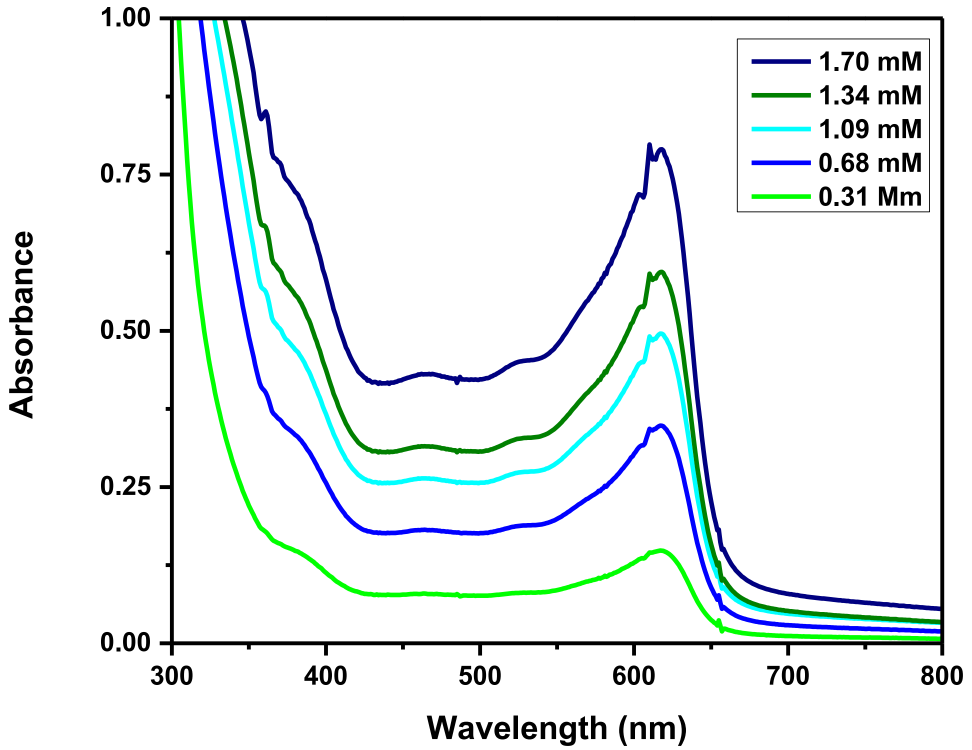

Figure S14. UV-Vis spectra of ferrocenium hexafluorophosphate with 0.1 M $[Bu_4N][PF_6]$ in DME at different concentrations.

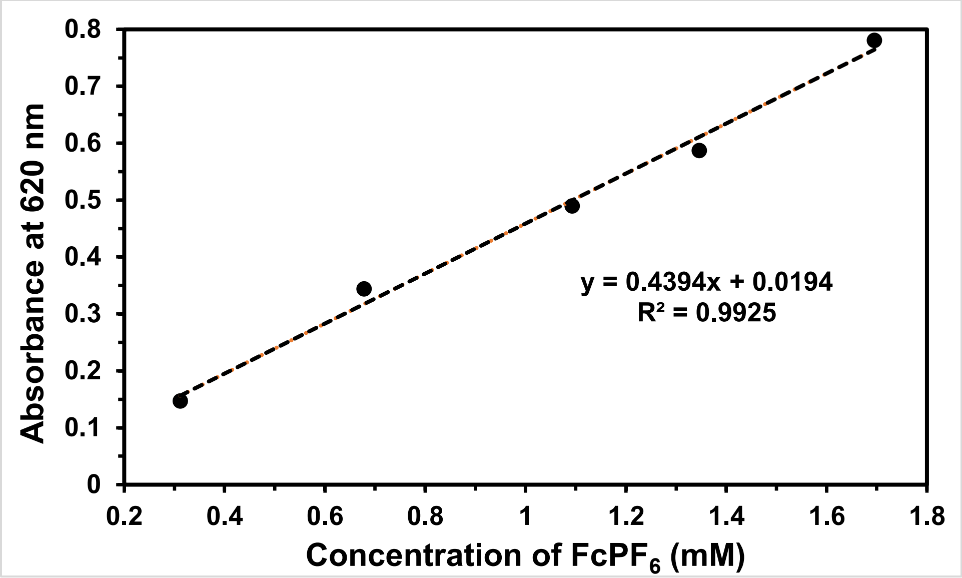

Figure S15. Calibration curve of absorbance at 620 nm versus concentration of $FcPF_6$. A linear regression was constructed giving a best fit line of y = 0.4394x + 0.0194.

S4.3 *Nido NMR Binding Procedure*

A stock solution of 7.64 mM $[Bu_4N]_2$[**^{14C4}Cb**] was prepared with 0.1 M $[Bu_4N][PF_6]$ in DME by bulk electrolysis of **^{14C4}Cb** (see S4.1). A stock solution of 8.3 mM $[Li(OEt_2)_{2.5}]$[TFAB]with 0.1 M $[Bu_4N][PF_6]$ in DME was prepared. The desired ratio of **$^{14C4}Cb^{2-}$** and $[Li(OEt_2)_{2.5}]$[TFAB] were dispensed into small vials using Hamilton syringes. The contents of the vial were mixed thoroughly and transferred to an NMR tube. ^{1}H, ^{7}Li and $^{11}B\{^{1}H\}$ NMR spectra were collected on a Bruker Avance NEO 500 MHz. ^{7}Li NMR spectra were collected with 128 scans and a 1 second T_1 relaxation delay. The example $^{11}B\{^{1}H\}$ NMR spectrum below displays more asymmetry than what was observed in Fig. S8 and we attribute this to the competing large excess of outer sphere $[Bu_4N]^+$ from the supporting electrolyte. Addition of a large excess of $[Li(OEt_2)_{2.5}]$[TFAB] yields a spectrum similar to Fig. S8.

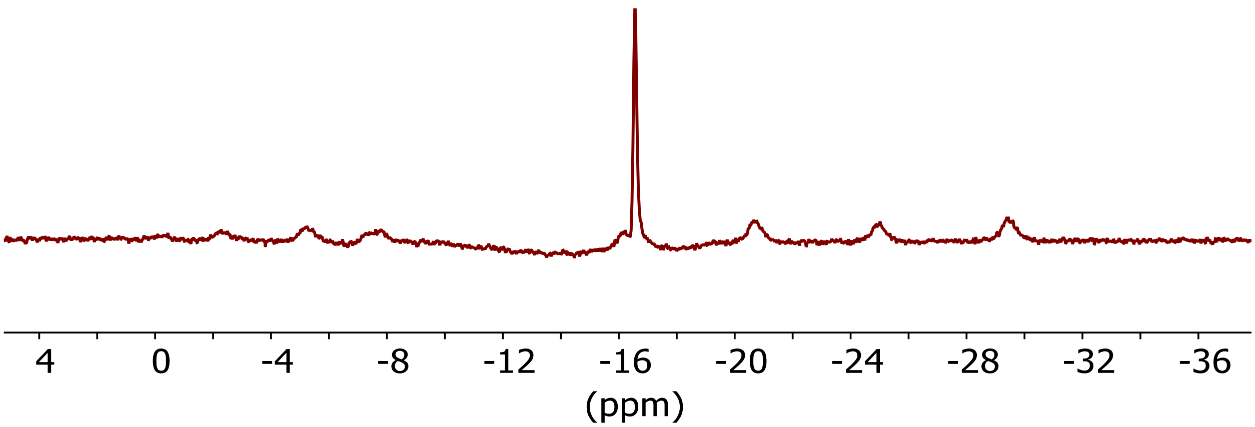

Figure S16. Example 160 MHz $^{11}B\{^1H\}$ NMR spectrum upon addition of 1 equiv. of $[Li(OEt_2)_{2.5}][TFAB]$ to ^{14C4}**Cb**$^{2-}$.

S5. Electrochemistry

S5.1 *Randles-Ševčík Analysis*

CVs of a 2.9 mM solution of ^{14C4}**Cb** in 0.1 M $[Bu_4N][PF_6]$ DME was taken at scan rates of: 50 mV/s, 100 mV/s, 250 mV/s, 350 mV/s, 500 mV/s. The electrodes consisted of a glassy carbon working electrode, Pt wire counter electrode, and Ag/AgOTf reference electrode. The working electrode was polished between each scan. The peak currents, $i_{p,c}$, and $i_{p,a}$, were taken at each scan rate after subtracting a straight line prior to the redox event using OriginPro 9 software. The peak current was plotted versus square root of scan rate. The data was fit to linear regressions, and the corresponding slope was used to calculate diffusion coefficients through the Randles-Ševčík equation (Equation 7). The corresponding diffusion coefficients are 7.02×10^{-7} cm^{2}s^{-1} and 1.77×10^{-7} cm^{2}s^{-1} for ^{14C4}**Cb** and ^{14C4}**Cb**$^{2-}$, respectively.

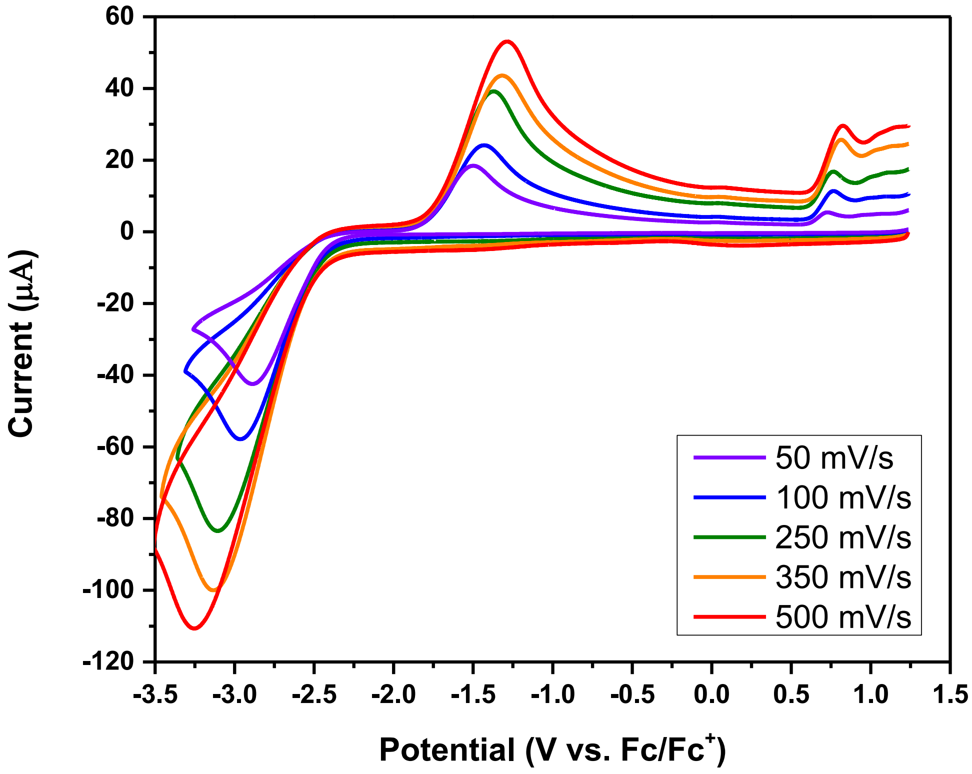

Figure S17. Variable scan rate of **^{14C4}Cb** in 0.1 M $[Bu_4N][PF_6]$ DME. Purple – 50 mV/s. Blue – 100 mV/s. Green – 250 mV/s. Orange – 350 mV/s. Red – 500 mV/s. Glassy carbon working electrode, Pt wire counter electrode, and Ag/AgOTf reference electrode.

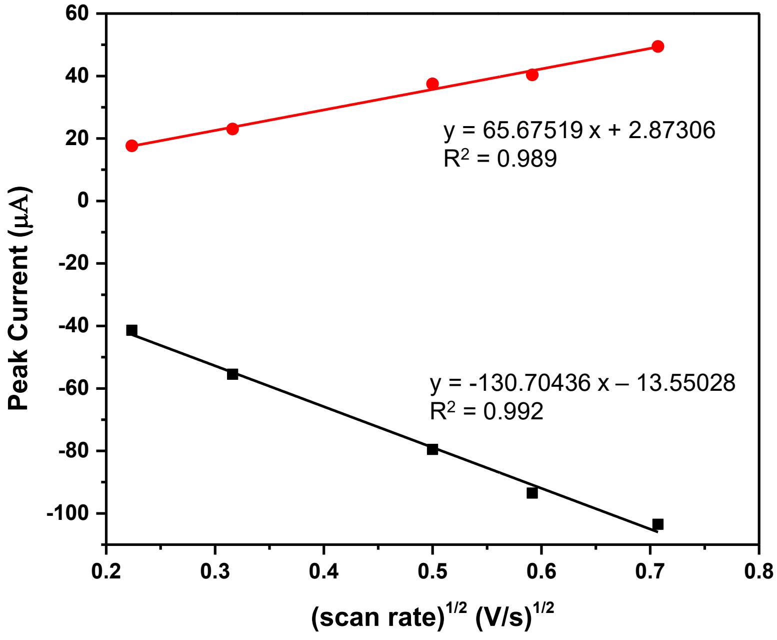

Figure S18. Graph of peak current versus square root of scan rate for Figure S17. Black squares – cathodic peak currents. Red circles – anodic peak currents.

S5.2 *CV Titration Procedure*

Stock solutions of 5.8 mM ^{14C4}**Cb**, 2.9 mM $[Li(OEt_2)_{2.5}][TFAB]$, 29 mM $NaPF_6$, and 250 mM KPF_6 were prepared in volumetric flasks with 0.1 M $[Bu_4N][PF_6]$ in DME in a nitrogen glovebox. Using a 500 µL Hamilton syringe, 500 µL of ^{14C4}**Cb** was dispensed into the electrochemical cell. Then, the corresponding metal solution was dispensed into the cell using a Hamilton syringe. Electrolyte solution was dispensed into the cell so that the final volume of solution had a total volume of 1 mL. Each solution was thoroughly stirred for 30 minutes. Stirring was discontinued and then the CV was taken at 50 mV/s using a freshly polished glassy carbon working electrode. All stock solutions, cell mixtures, and corresponding CV measurements were performed in triplicate. The peak current of each oxidation, $i_{p,a}$, was recorded after a straight line subtraction using OriginPro 9 software. The peak current of 2.9 mM ^{14C4}**Cb** with no metal, 17.69 µA, was determined by CV with 6 trial averages and was used for all Δi calculations. The average $\log(\Delta i/i_{p,a})$ versus metal concentration was plotted for each alkali metal with the standard deviation shown as error bars. A linear regression with no weighting was used on OriginPro 9. The y-intercept and error correspond to the log K for each metal. The slope and error of each linear regression corresponds to the stoichiometry.

S5.3 *CVs of* ^{14C4}***Cb*** *with* Li^+*,* Na^+*, or* K^+

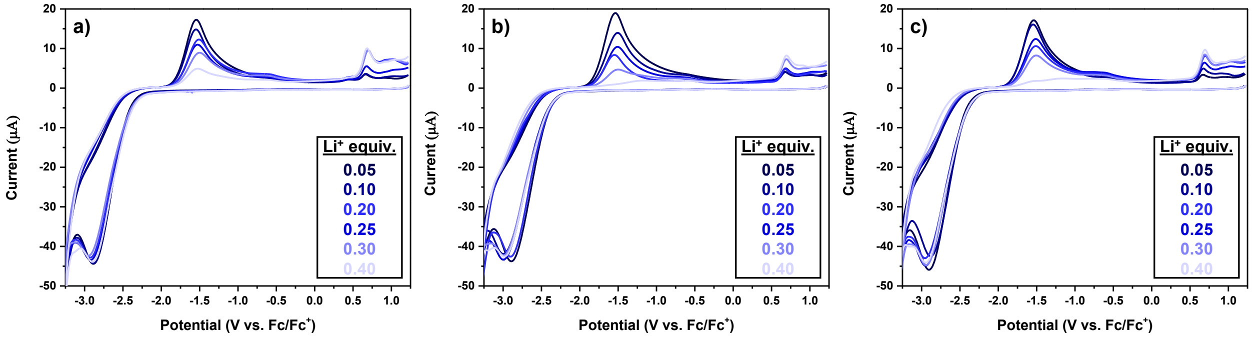

Figure S19. CVs of 2.9 mM ^{14C4}**Cb** with different equivalents of Li^+. Concentrations of $[Li(OEt_2)_{2.5}][TFAB]$ added: 0.145 mM, 0.29 mM, 0.58 mM, 0.725 mM, 0.87 mM, 1.16 mM. a) Trial 1 b) Trial 2 c) Trial 3

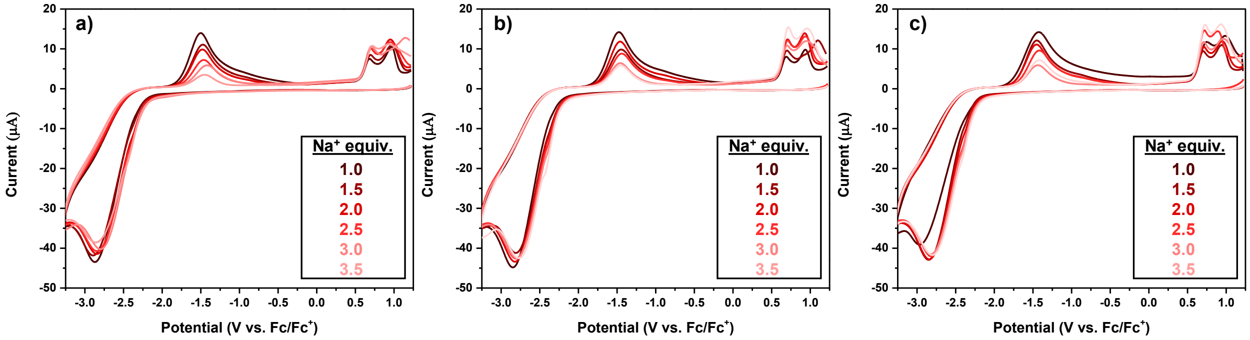

Figure S20. CVs of 2.9 mM ^{14C4}**Cb** with different equivalents of Na^+. Concentrations of $NaPF_6$ added: 2.9 mM, 4.35 mM, 5.8 mM, 7.25 mM, 8.7 mM, 10.15 mM. a) Trial 1 b) Trial 2 c) Trial 3

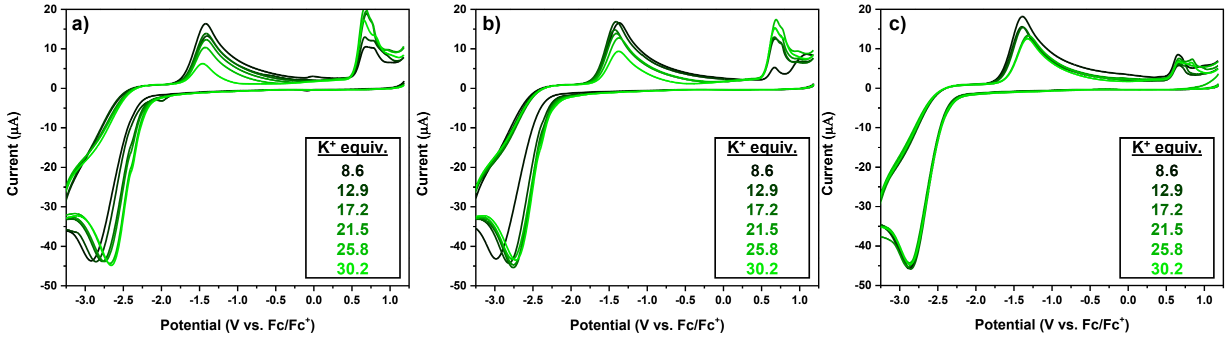

Figure S21. CVs of 2.9 mM ^{14C4}**Cb** with different equivalents of K^+. Concentrations of KPF_6 added: 25 mM, 37.5 mM, 50 mM, 62.5 mM, 75 mM, 87.5 mM. a) Trial 1 b) Trial 2 c) Trial 3

S5.3 *Binding constant equation*

Denoting $^{14C4}\mathbf{Cb}^{2-}$ as Cb^{nido} and the alkali metal cations as M, we have a general binding equilibrium where x metal centers bind to each Cb^{nido} center to generate the bound metal-carborane species, denoted as $\left[Cb^{nido}\ xM\right]$. We thus have the equilibrium,

$$Cb^{nido} + x\,M \rightleftarrows \left[Cb^{nido}\ xM\right] \qquad \text{(S1)}$$

$$K = \frac{[Cb^{nido}\ xM]}{[Cb^{nido}][M]^x} \qquad \text{(S2)}$$

Rearranging Equation S2, we have:

$$\frac{[Cb^{nido}\ xM]}{[Cb^{nido}]} = K[M]^x \qquad \text{(S3)}$$

Applying a logarithm function on both sides of this equation leads to:

$$log\ \frac{[Cb^{nido}\ \ xM]}{[Cb^{nido}]} = x\ log[M] + \log K \qquad \text{(S4)}$$

Assuming rapid and complete conversion of *closo→nido* species near the working electrode, the sum of $\left[Cb^{nido}\ xM\right]$ and $\left[Cb^{nido}\right]$ is constant and equal to the initial bulk concentration of $^{14C4}\mathbf{Cb}$, denoted as $[Cb^{closo}]_o$ as expressed in Equation S5.

$$[Cb^{closo}]_o = \left[Cb^{nido}\ xM\right] + [Cb^{nido}] \qquad \text{(S5)}$$

which can be rearranged and substituted in equation (S4) to obtain:

$$log\ \frac{[Cb^{closo}]_o - [Cb^{nido}]}{[Cb^{nido}]} = xlog[M] + \log K \qquad \text{(S6)}$$

The *nido→closo* peak anodic current ($i_{p,a}$) in the CVs of $^{14C4}\mathbf{Cb}$ has a linear dependence on the concentration of $^{14C4}\mathbf{Cb}^{2-}$ and thus enables measuring the $[Cb^{nido}]$ that remains unbound to alkali cations. We further note that the *nido→closo* peak oxidation current is diffusion-limited and adheres to the Randles-Ševčík equation (Equation S7, Figure S18), where: *n* is the number of electrons transferred, *F* is Faraday's constant, *A* is the area of the electrode, *D* is the diffusion coefficient, *v* is the scan rate, *R* is the gas constant, *T* is temperature and *C* is the bulk concentration of the redox probe.

$$i_{p,a} = 0.4463nFA\left(\frac{nFvD}{RT}\right)^{\frac{1}{2}} C = p \cdot [Cb^{nido}] \qquad (S7)$$

We held all the terms in the Randles-Ševčík equation constant besides the concentration of the redox probe in the CVs we have acquired; hence, the peak oxidation current is directly proportional to $[Cb^{nido}]$ ($C = [Cb^{nido}]$). Substituting Equation S7 into Equation S6, we find:

$$log\ \frac{i_o - i_{p,a}}{i_{p,a}} = log\ \frac{\Delta i}{i_{p,a}} = \ x\ log[M] + \log K \qquad (S8)$$

where i_o is the peak oxidation current in absence of any metals, and $i_{p,a}$ is the oxidation current once metal is added, and Δi is the magnitude of current decrease upon addition of Li^+, Na^+ or K^+.[4-7] Plotting the logarithm of the relative current change log $(\frac{\Delta i}{i_{p,a}})$ with respect to the concentration of metal, the y-intercept would be equal to log *K*, while the number of metal ions binding to each **$^{14C4}Cb^{2-}$** would equal the slope (see section S5.4 below). The obtained log *K* for each metal will be the summation of each individual alkali metal binding event to **$^{14C4}Cb^{2-}$** (*e.g.*, log K_1 + log K_2).

S5.4 *CV titration linear regressions*

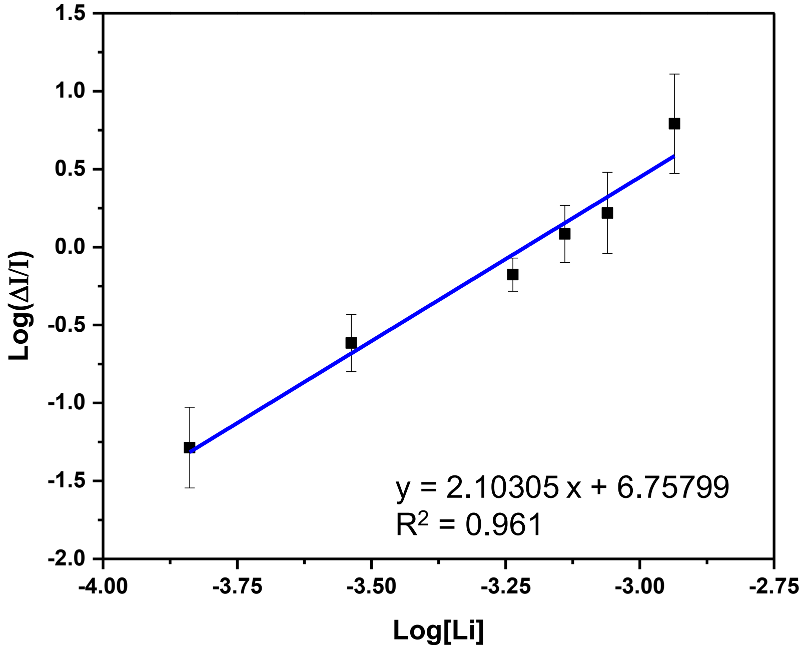

Figure S22. Plot of Log(ΔI/I) versus Log[Li]. *K* is 6.76 ± 0.62. Li stoichiometry is 2.10 ± 0.19.

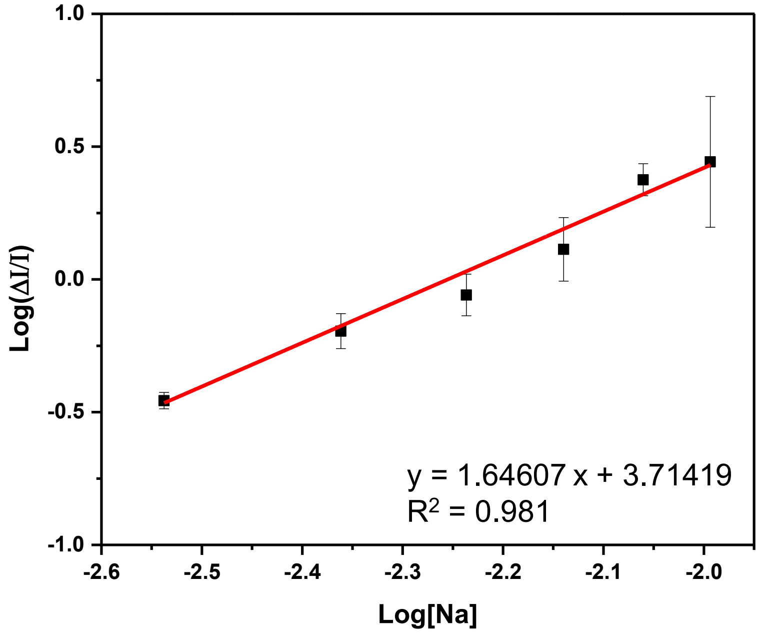

Figure S23. Plot of Log(ΔI/I) versus Log[Na]. *K* is 3.71 ± 0.25. Na stoichiometry is 1.65 ± 0.10.

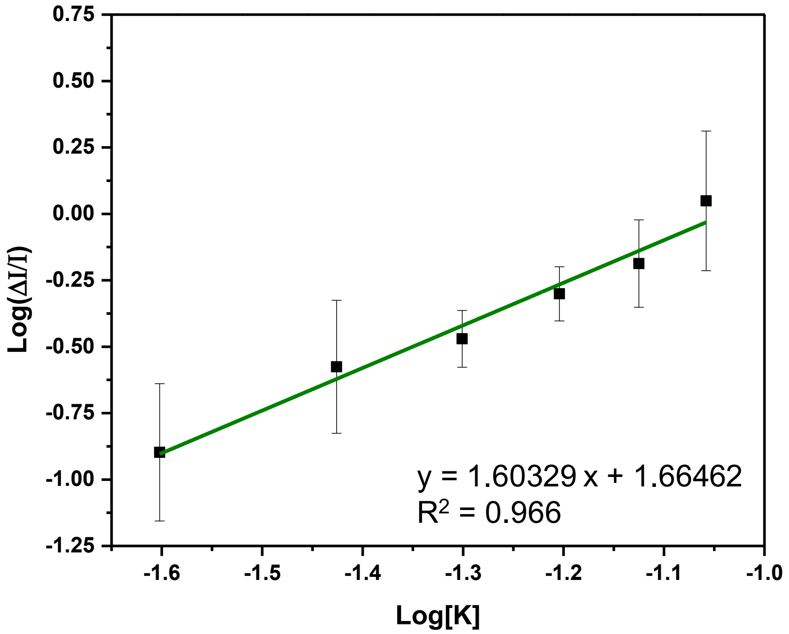

Figure S24. Plot of Log(ΔI/I) versus Log[K]. *K* is 1.66 ± 0.17. K stoichiometry is 1.60 ± 0.13.

S5.5 *CV Controls*

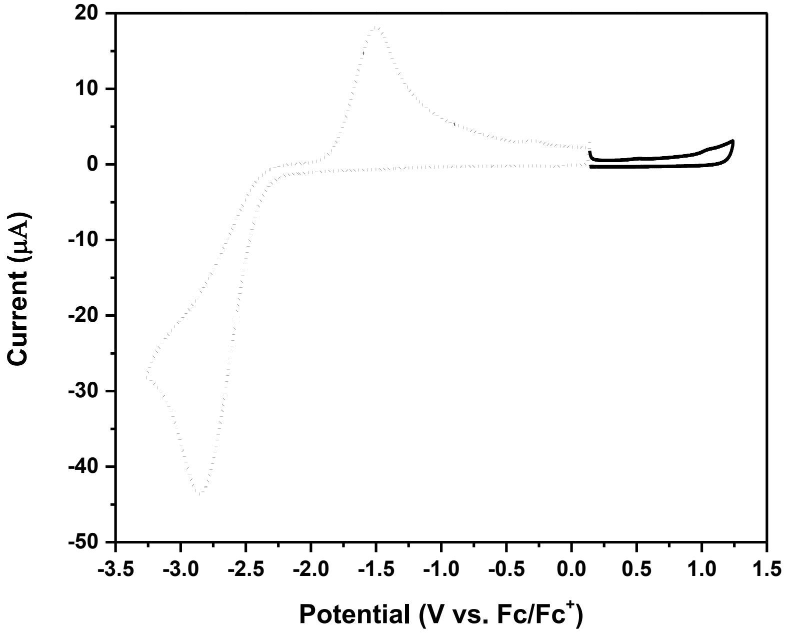

Figure S25. Dotted trace – CV of 2.9 mM **^{14C4}Cb** in 0.1 M $[Bu_4N][PF_6]$ DME. Solid trace – Anodic scan of the same used electrode in a fresh M $[Bu_4N][PF_6]$ DME solution.

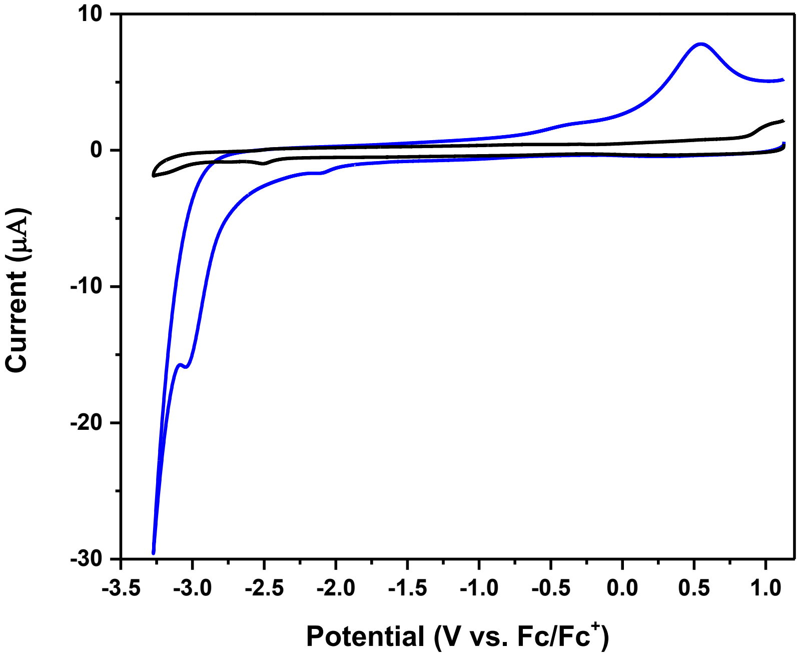

Figure S26. CV of 5.8 mM $[Li(OEt_2)_{2.5}]$[TFAB] in 0.1 M $[Bu_4N][PF_6]$ DME. Black – blank. Blue – with $[Li(OEt_2)_{2.5}]$[TFAB].

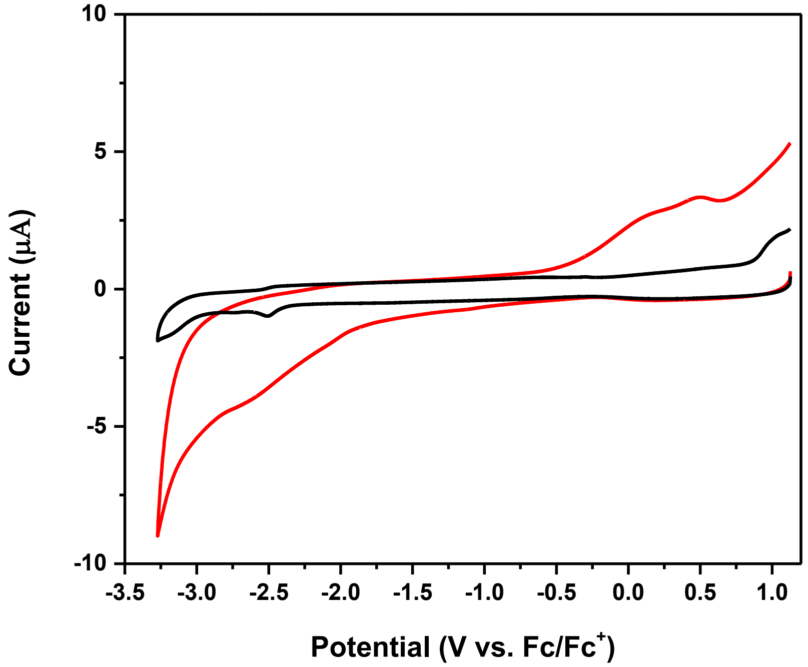

Figure S27. CV of 5.8 mM $NaPF_6$ in 0.1 M $[Bu_4N][PF_6]$ DME. Black – blank. Red – with $NaPF_6$.

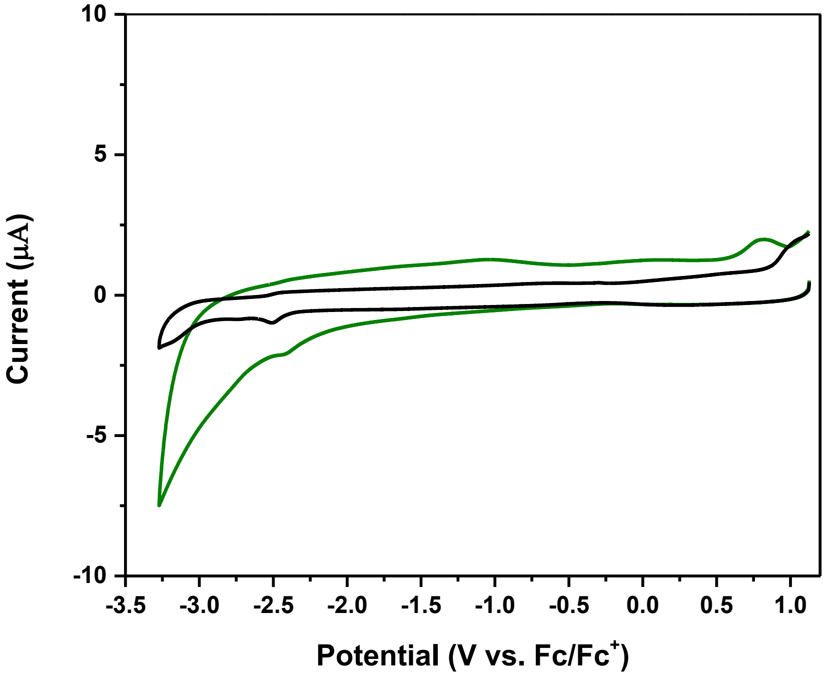

Figure S28. CV of 5.8 mM KPF_6 in 0.1 M $[Bu_4N][PF_6]$ DME. Black – blank. Green – with KPF_6.

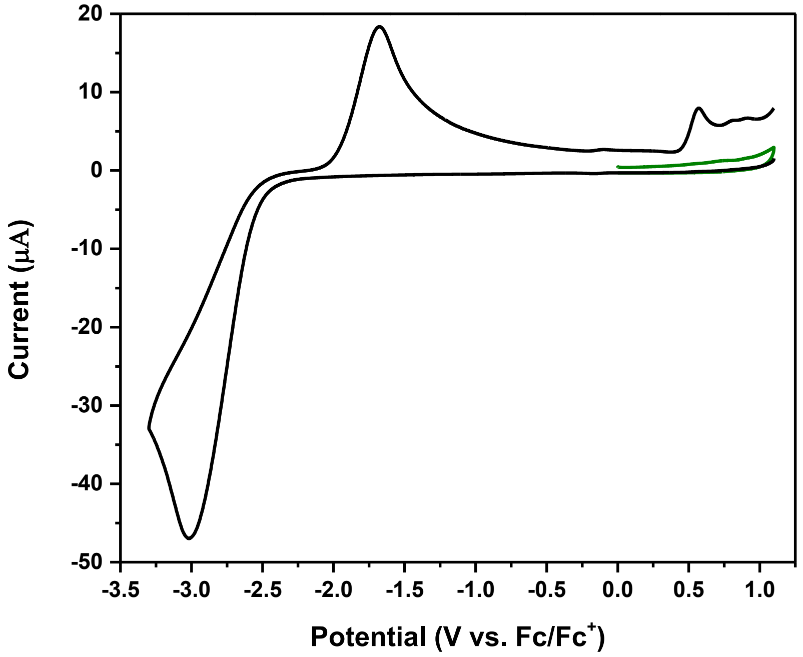

Figure S29. Black – cathodic CV of 2.9 mM **^{14C4}Cb** in 0.1 M [Bu_4N][PF_6] DME. Green – anodic CV scan of 2.9 mM **^{14C4}Cb** in 0.1 M [Bu_4N][PF_6] DME.

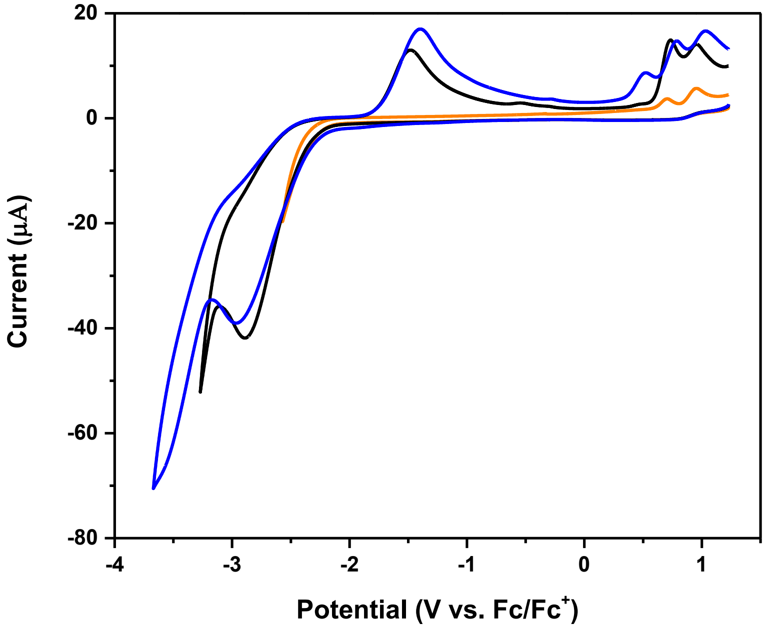

Figure S30. CVs of 2.9 mM **^{14C4}Cb** and 0.87 mM [Li(OEt_2)$_{2.5}$][TFAB] in 0.1 M [Bu_4N][PF_6] DME with different cathodic potential window cut offs. Orange – -2.57 V. Black – -3.27 V. Blue – -3.67 V.

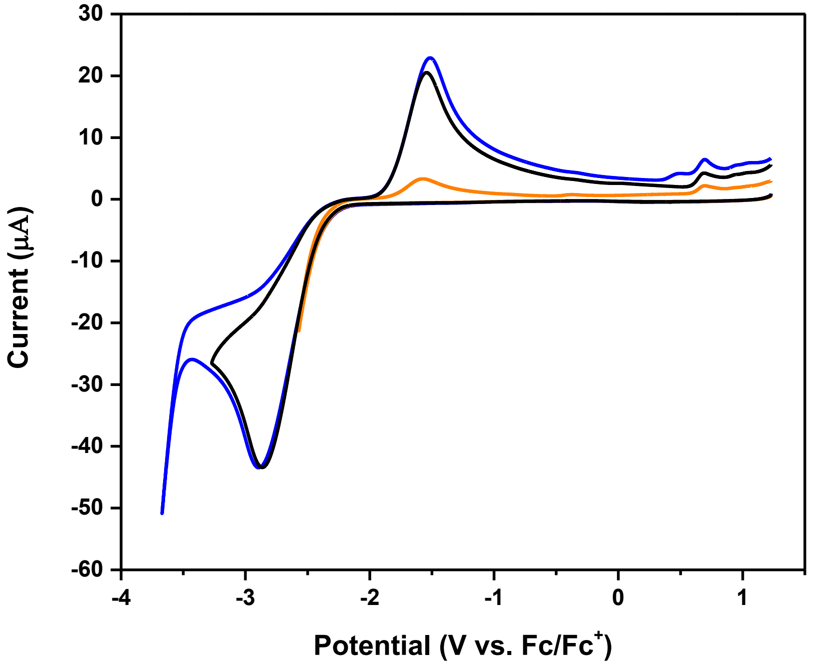

Figure S31. CVs of 2.9 mM **[14C4]Cb** in 0.1 M $[Bu_4N][PF_6]$ DME with different cathodic potential window cut offs. Orange – -2.57 V. Black – -3.27 V. Blue – -3.67 V.

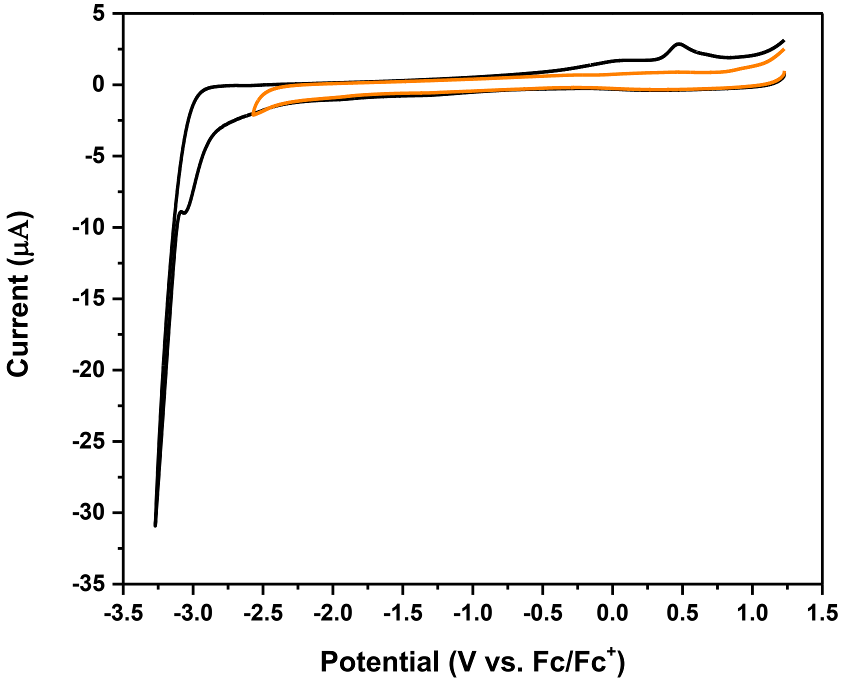

Figure S32. CVs of 0.87 mM $[Li(OEt_2)_{2.5}][TFAB]$ in 0.1 M $[Bu_4N][PF_6]$ DME with different cathodic potential window cut offs. Orange – -2.57 V. Black – -3.27 V.

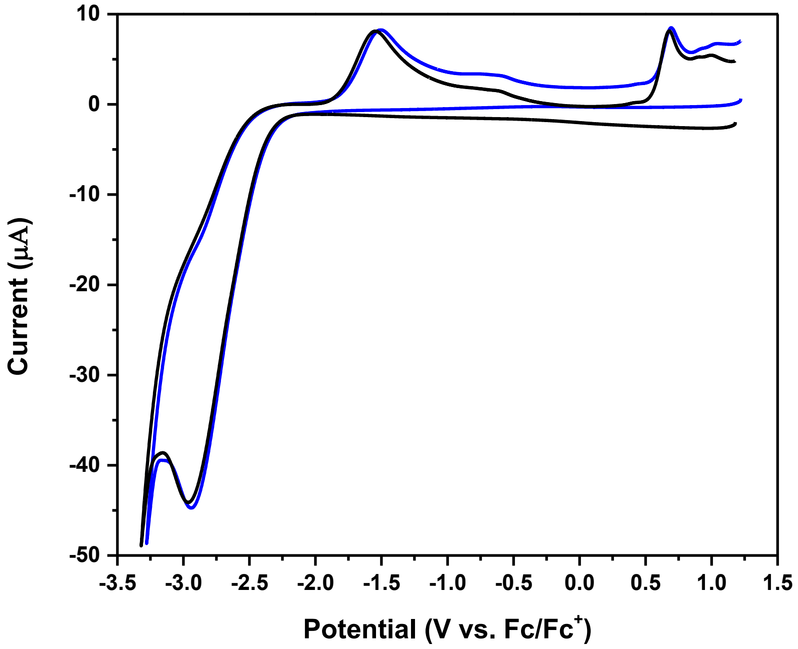

Figure S33. CVs of 2.9 mM **^{14C4}Cb** in 0.1 M [Bu_4N][PF_6] with Li^+ ([$Li(OEt_2)_{2.5}$][TFAB]) and 12-crown-4. Blue – 0.87 mM Li^+ Black – 0.87 mM Li^+ and 1.74 mM 12-crown-4.

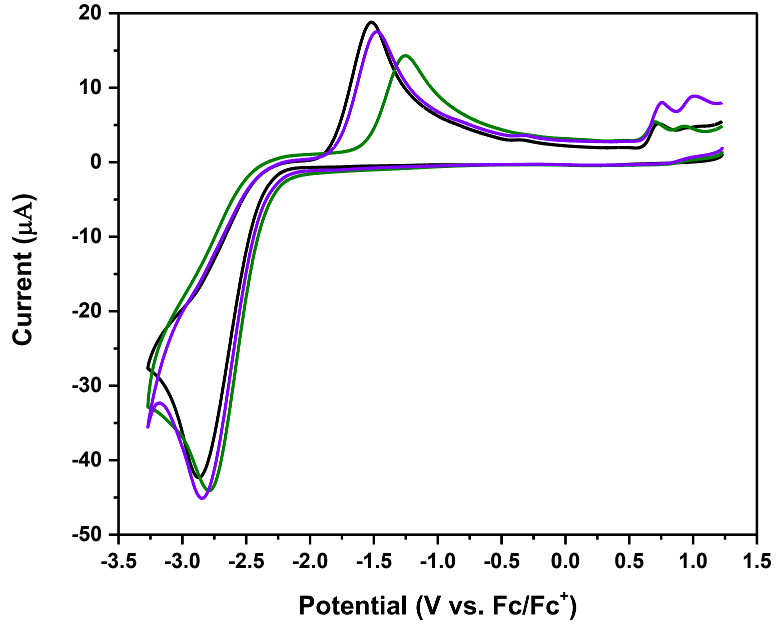

Figure S34. CVs of 2.9 mM **^{14C4}Cb** in 0.1 M [Bu_4N][PF_6] with K^+ (KPF_6) and/or 18-crown-6. Black – with 30 equivalents of 18-crown-6. Green – with 30 equivalents of K^+. Purple – with 30 equivalents of 18-crown-6 and 30 equivalents of K^+.

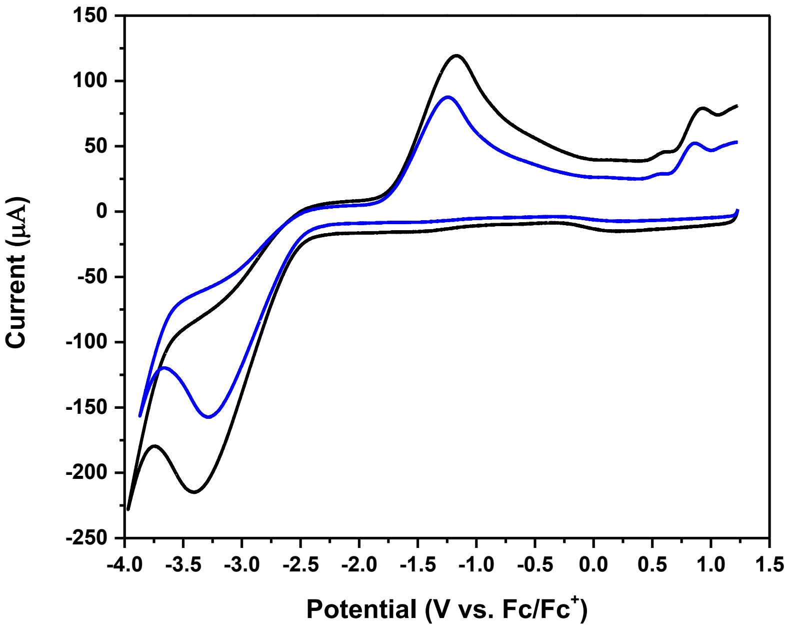

Figure S35. CVs of 2.9 mM **^{14C4}Cb** in 0.1 M $[Bu_4N][PF_6]$ at fast scan rates: Black – 2V/s. Blue – 1 V/s.

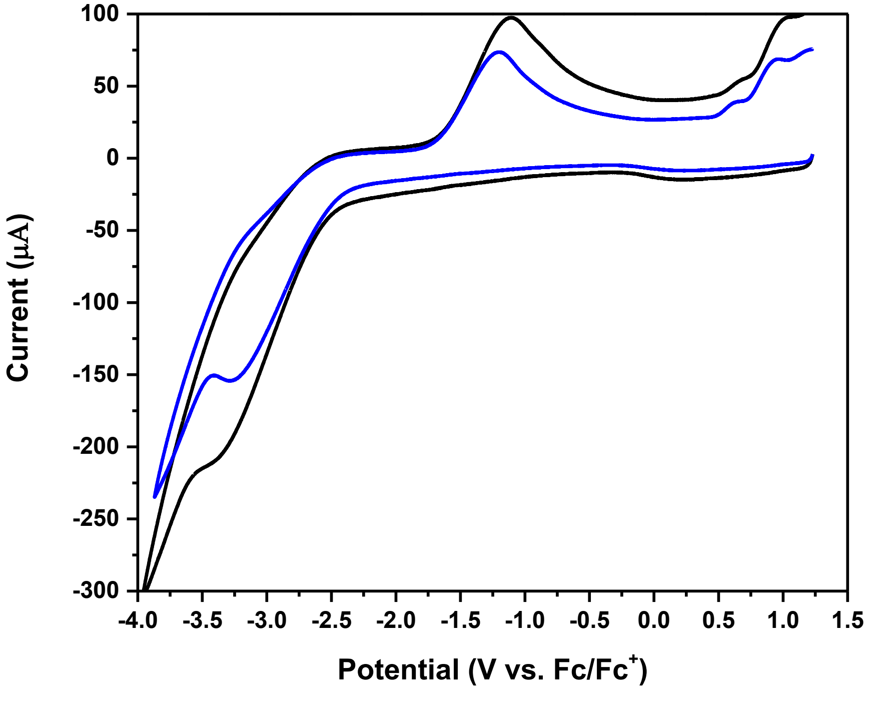

Figure S36. CV of 2.9 mM **^{14C4}Cb** in 0.1 M $[Bu_4N][PF_6]$ with 0.87 mM $[Li(OEt_2)_{2.5}]$[TFAB] at fast scan rates: Black – 2V/s. Blue – 1 V/s.

S6. Simulated Voltammetry

S6.1 *Simulation General Parameters*

All simulations were performed using DigiSim version 3.03b. Pre-equilibration was enabled. A semi-infinite model for diffusion was used. A planar electrode with surface area 0.07071 cm^2 was used. The capacitance double layer set to 1 × 10^{-5} F. The temperature was set to the same temperature as the experiments, 295 K. The resistance in the cell with 0.1 M $[Bu_4N][PF_6]$ DME was measured by the peak to peak separation of the ferrocene/ferrocenium redox couple at 500 mV/s (3600 Ω) and used for all simulations. The concentration of ^{14C4}**Cb** was set to 2.9 mM. The CV was scanned from 1.25 to -3.25 and back with a potential step of 0.001 V and a scan rate of 50 mV/s unless otherwise stated.

CVs of ^{14C4}**Cb** were simulated at 50 mV/s, 250 mV/s, and 500 mV/s to obtain fits for the remaining parameters (Figure S36). To simulate the metal-free carborane species, an ECEC mechanism was implemented to account for the chemical changes (*i.e.*, cage rearrangement) of carborane between its *closo* and *nido* forms (Figure S36). ^{14C4}**Cb** is represented by *closo* and *closo*$^{2-}$ is the reduced species before bond cleavage and cage rearrangement. ^{14C4}**Cb**$^{2-}$ is denoted as *nido*$^{2-}$ and *nido*^{0} is the neutral species before C–C bond formation and carborane cage reformation.

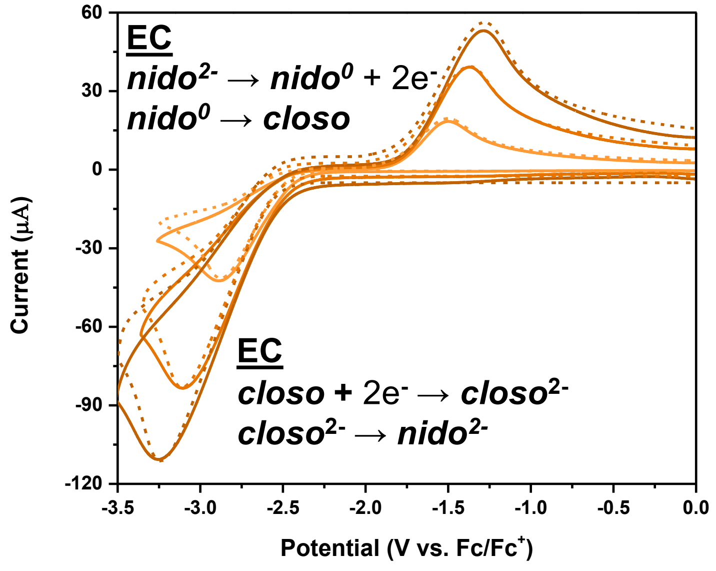

Figure S37. Experimental and simulated CVs of 2.9 mM ^{14C4}**Cb** in 0.1 M $[Bu_4N][PF_6]$ DME following an ECEC mechanism. Solid – experimental. Dashed – simulated. Scan rates are 50 mV/s, 250 mV/s and 500 mV/s.

A diffusion coefficient of 8×10^{-6} cm^2/s was used for *closo* and *closo*$^{2-}$. A diffusion coefficient of 1×10^{-6} cm^2/s was used for *nido*$^{2-}$ and *nido*^{0}. We note that the diffusion coefficients were experimentally determined in section S5.1, but the values used here provided a better fit to the experimental data. For the first electron transfer step (*closo* + 2 e^- → *closo*$^{2-}$), the following parameters were used: heterogenous electron transfer rate ($k_o = 5 \times 10^{-5}$ cm/s); heterogenous charge transfer coefficient ($\alpha = 0.20$); an initial reduction potential (E_o = -2.48 V). For the second electron transfer step (*nido*$^{2-}$ → *nido*^{0} + 2 e^-), the following parameters were used: $k_o = 5 \times 10^{-5}$ cm/s; $\alpha = 0.85$; E_o = -1.78 V. For the chemical changes, (*closo*$^{2-}$ → *nido*$^{2-}$) and (*nido*^{0} → *closo*), we assumed fast kinetics with a $k_f = 1 \times 10^5$ and favorable thermodynamics with a K_{eq} of 1×10^{10} to ensure complete chemical conversion.

S6.2 *CV Simulations*

For the metal CV simulations, the parameters as described in section S6.1 were used, with the exception of reduction potentials E_o which were optimized for each scan, and the cathodic heterogenous charge transfer coefficient α was kept constant at 0.15. Literature values for diffusion coefficients of Li^+ (1.5×10^{-5} cm^2/s), Na^+ (1.0×10^{-5} cm^2/s), and K^+ (9.0×10^{-6} cm^2/s) were used.[8-10] An ECCCEC mechanism was implemented with two additional competing chemical reactions occurring between *nido*$^{2-}$ and the alkali metal, M^+ to form M*nido*$^-$ and M_2*nido* (Figure 8a). The metal binding events must occur sequentially as DigiSim 3.03b is not capable of simulating two concurrent binding events. The diffusion coefficient of *nido* (1×10^{-6} cm^2/s) was used for the metal-carborane species. The voltammetry was then simulated with different concentrations of Li^+, Na^+ or K^+ and fit to experimental data (Figures S37-S39). The voltammetry was fit to experimental data to obtain K_{eq} values for each metal binding to **$^{14C4}Cb^{2-}$**. Note: the simulated cathodic event does not vary with different equivalents of metal resulting in superimposed dashed lines for the observed reduction. We note that via simulations, we cannot converge on separate log *K* values with confidence and have higher regard for the summed total log *K*.

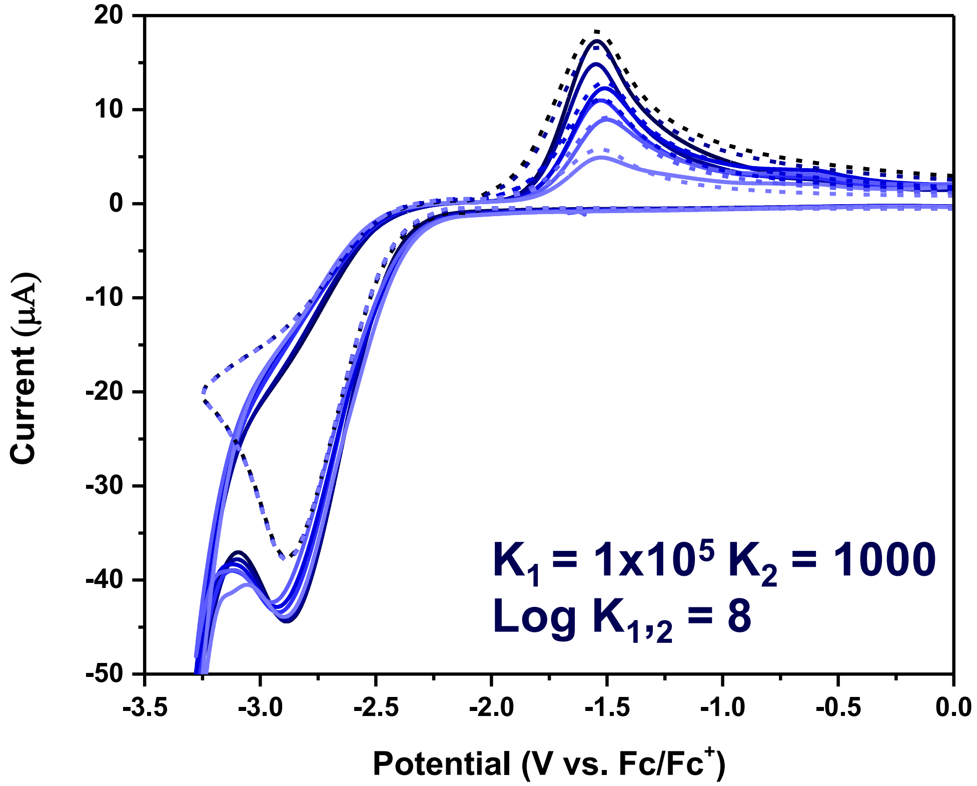

Figure S38. Simulated (dashed) and experimental (solid) CVs of **^{14C4}Cb** with different concentrations of Li^+ (0.145 mM, 0.29 mM, 0.58 mM, 0.725 mM, 0.87 mM, 1.16 mM). Simulated using a k_{f1} of 300, k_{f2} of 1, K_1 of 1 x 10^5 and K_2 of 1000.

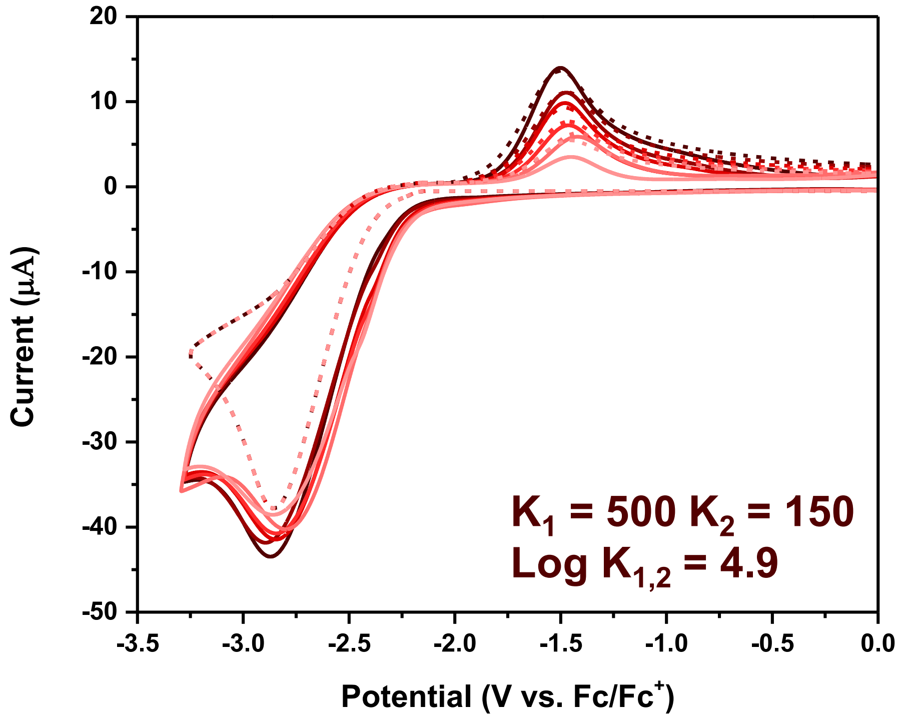

Figure S39. Simulated (dashed) and experimental (solid) CVs of **^{14C4}Cb** with different concentrations of Na^+ (2.9 mM, 4.35 mM, 5.8 mM, 7.25 mM, 8.7 mM, 10.15 mM). Simulated using a k_{f1} of 5, k_{f2} of 1.5, K_1 of 500 and K_2 of 150.

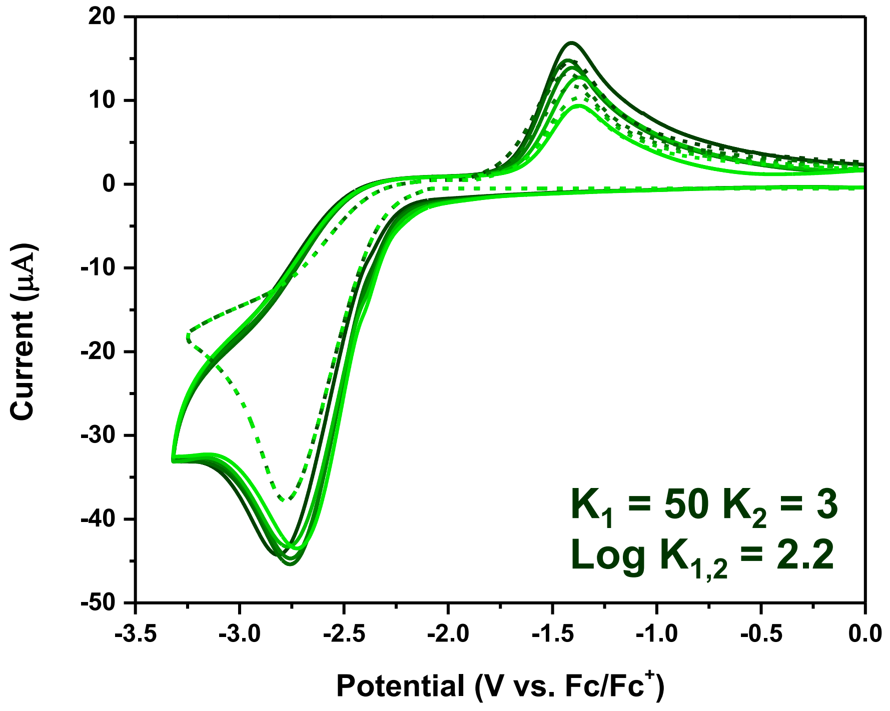

Figure S40. Simulated (dashed) and experimental (solid) CVs of **^{14C4}Cb** with different concentrations of K^+ (37.5 mM, 50 mM, 62.5 mM, 75 mM, 87.5 mM). Simulated using k_{f1} of 0.3 k_{f2} of 0.1, K_1 of 50 and K_2 of 3.

S6.3 *CV Simulation Controls*

Using the same parameters as reported in section S6.2 unless otherwise stated, we simulated **^{14C4}Cb** with 0.87 mM [Li(OEt$_2$)$_{2.5}$][TFAB] at fast scan rates of 1 V/s and 2 V/s where Li^+ is no longer fully depleted near the electrode. The increased peak to peak separation at high scan rates was better modeled by a heterogenous charge transfer rate k_o of 1×10^{-4} for the *closo* and *nido* redox events. The CV was scanned from 1.25 V to -3.87 V or -3.93 V and back with a potential step of 0.001 V and a scan rate of 1 V/s or 2 V/s respectively. Additionally, we simulated **^{14C4}Cb** with Li^+ with analogous conditions as reported in section S6.2 except for increasing the diffusion coefficient of Li^+ by a hypothetical factor of 10 (1.5×10^{-4}) to demonstrate that the chemical reaction is limited by mass transport of Li^+, and not the log *K* binding constant. Under these conditions, only 0.25 equiv. of Li^+ fully suppresses the *nido* oxidation.

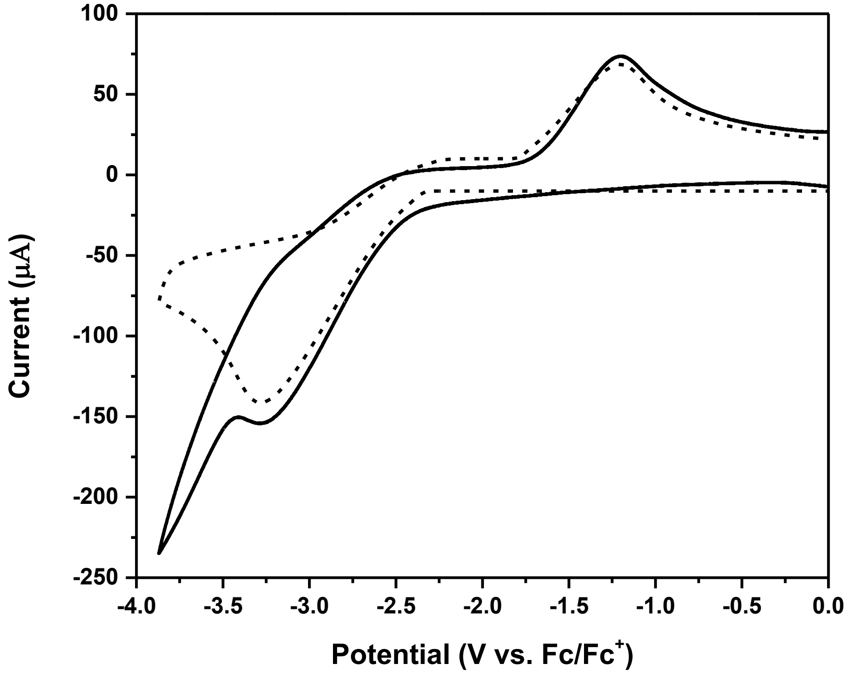

Figure S41. Simulated (dashed) and experimental (solid) CVs of **^{14C4}Cb** with 0.87 mM of Li^+ at 1 V/s.

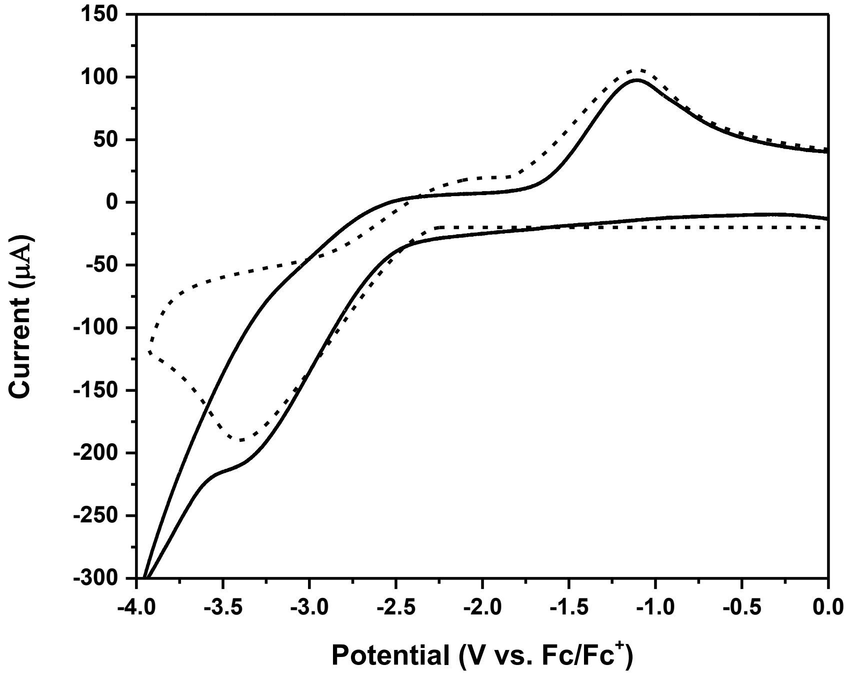

Figure S42. Simulated (dashed) and experimental (solid) CVs of **^{14C4}Cb** with 0.87 mM of Li^+ at 2 V/s.

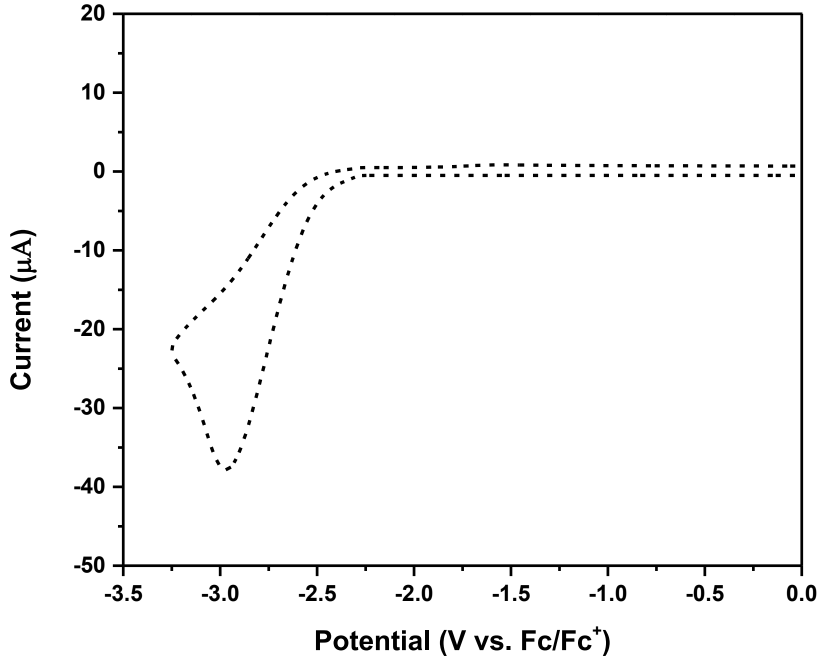

Figure S43. Simulated (dashed) CV of **^{14C4}Cb** with 0.725 mM of Li^+ with a hypothetical increased Li^+ diffusion constant of 1.5×10^{-4}.

S7. Supplemental Figure

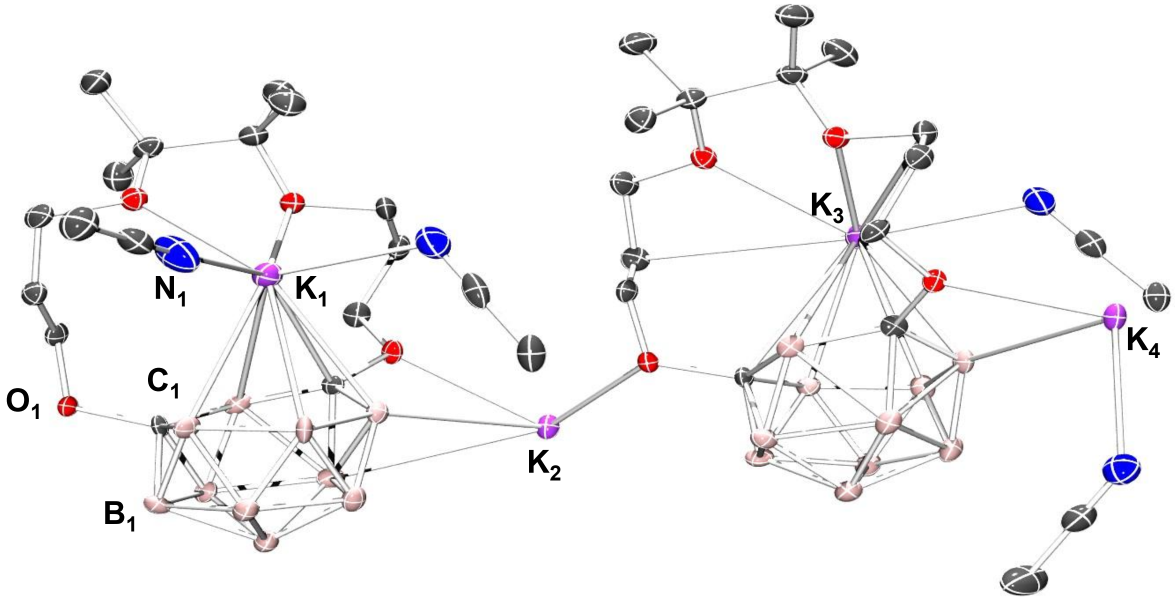

Figure S44. Solid-state molecular structure of $\mathbf{K_2{}^{14C4}Cb}$ obtained by single crystal X-ray diffraction. The repeating units of $\mathbf{K_2{}^{14C4}Cb}$ are displayed. H atoms and uncoordinated co-crystallized solvent molecules are omitted for clarity.

S8. DFT Studies

DFT calculations were performed using Gaussian 09.2.[11] Geometry optimization of all the molecules was carried out using the B3LYP-D3 method with Ahlrichs' def2-SVP basis set.[12-16] The solvation in acetonitrile was calculated using SMD.[17] Thermal energy corrections were extracted from the results of frequency analysis performed at the same level of theory. Frequency analysis of all the molecules and intermediates contained no imaginary frequency showing that these are energy minima.

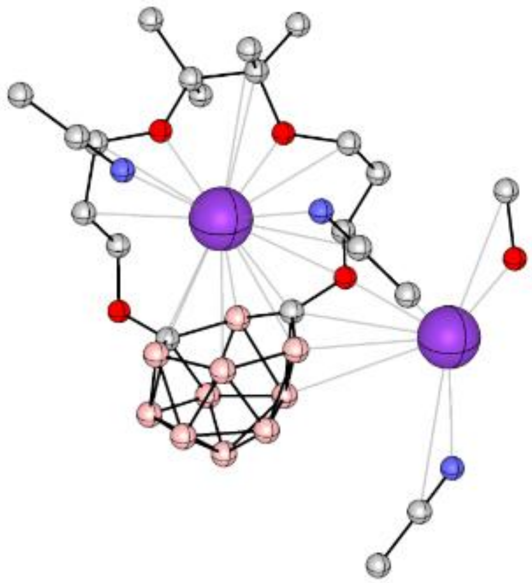

$^{mod}K_2{}^{14C4}Cb$:

K	3.80140	1.88410	-0.65640
K	-1.04390	-0.15930	1.07810
O	3.34020	4.20490	0.60630
O	1.44450	0.77560	-1.30070
O	-3.39750	-0.33600	-0.41710
O	-0.94940	-3.61680	-1.28160
O	-1.77770	1.67250	-0.85580
C	-2.08980	-2.79410	-1.46200
H	-2.65050	-3.22260	-2.31050
H	-1.78750	-1.77810	-1.76440
C	1.34270	-0.49310	-0.72560
C	0.18920	-3.00600	-0.68970
N	0.17620	1.90190	2.64190
C	0.59280	1.16800	-2.38670
H	-0.25880	0.48140	-2.46040
H	1.15850	1.11200	-3.33310
C	-3.93890	0.72660	-1.23440
C	-3.01360	-2.70290	-0.24480
H	-3.52920	-3.66450	-0.08550
H	-2.42860	-2.52060	0.66870
N	-2.96890	-0.81240	3.06130
C	-4.05430	-1.60290	-0.41790
H	-4.59940	-1.77010	-1.36240
H	-4.79940	-1.64220	0.39620
C	-0.75810	2.67110	-0.85660
H	-0.10920	2.49310	0.01770
H	-1.16530	3.68590	-0.74200
C	-3.17430	2.03280	-0.78590
C	0.07620	2.57680	-2.13210
H	-0.53250	2.87420	-3.00240
H	0.91130	3.29660	-2.06260

C	1.26100	2.13020	2.98060
C	-3.47970	3.20420	-1.73160
H	-3.09720	4.14820	-1.31800
H	-4.56230	3.33160	-1.86530
H	-3.02140	3.05310	-2.71810
C	-5.44800	0.85360	-0.98080
H	-5.98400	-0.00370	-1.41240
H	-5.85940	1.75930	-1.44670
H	-5.66700	0.88350	0.09590
C	-3.65710	0.41640	-2.71000
H	-2.57740	0.44930	-2.90930
H	-4.16400	1.13260	-3.37240
H	-4.03000	-0.58500	-2.96860
C	-4.07750	-1.12470	3.18810
C	6.36880	-0.82200	-0.31810
C	2.63090	2.40440	3.37870
H	3.08240	3.11430	2.66810
H	3.20660	1.46600	3.37240
H	2.65080	2.83490	4.39130
B	1.65360	-3.22760	-1.52830
H	1.67730	-3.71710	-2.62950
C	-5.47040	-1.51540	3.32280
H	-6.10250	-0.84340	2.72250
H	-5.77830	-1.45450	4.37780
H	-5.60170	-2.54870	2.96760
C	-3.51650	2.42590	0.65790
B	1.24930	-4.18650	-0.12350
H	0.96270	-5.34660	-0.29730
B	1.51340	-1.95910	1.69910
H	1.51230	-1.63700	2.87950
B	2.07020	-0.66990	0.59000
H	2.64920	0.30940	1.02940
C	6.74810	-2.22030	-0.22880
H	6.99190	-2.47460	0.81370
H	7.62440	-2.41350	-0.86620
H	5.90560	-2.84480	-0.56260
B	0.72830	-1.66750	-1.48440
H	0.31920	-1.50080	-2.60670
B	3.04420	-2.15890	0.85740
H	4.13870	-1.93360	1.32870
B	2.66570	-1.76490	-0.96540
H	3.51740	-1.34210	-1.71210
B	2.88080	-3.45300	-0.32570
H	3.86350	-4.07760	-0.65360
B	0.37560	-3.04080	0.96830
H	-0.55460	-3.53790	1.57560
B	2.09260	-3.56750	1.28960
H	2.47110	-4.36420	2.12530
H	-4.52070	2.86950	0.72560
H	-3.48110	1.55880	1.33290
H	-2.79750	3.17950	1.01430

C	2.10670	4.92530	0.58930
H	1.30030	4.20480	0.39610
H	1.90600	5.41330	1.55890
H	2.09270	5.69070	-0.20670
H	4.04920	4.81340	0.87090
N	6.04940	0.28940	-0.39470

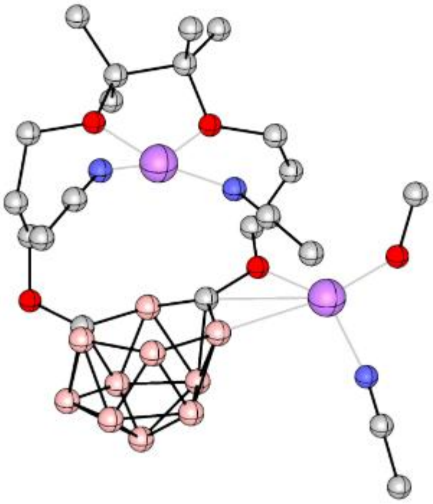

$^{mod}Li_2{}^{14C4}Cb$:

O	-2.87190	-3.41700	0.95250
O	-1.35050	-1.03800	-1.06720
O	3.18000	0.28100	-0.17010
O	0.79080	3.45630	-1.66660
O	1.76880	-1.80630	-0.35880
C	1.97860	2.68010	-1.62920
H	2.65560	3.12650	-2.37730
H	1.77040	1.64790	-1.95580
C	-1.32260	0.32190	-0.70880
C	-0.34850	2.87590	-1.04460
N	0.17960	-1.14540	2.21180
C	-0.50190	-1.55050	-2.10700
H	0.33400	-0.85880	-2.25580
H	-1.07410	-1.60690	-3.04850
C	3.84280	-0.76950	-0.92550
C	2.65600	2.64430	-0.26180
H	3.08530	3.63190	-0.02230
H	1.90200	2.43750	0.50580
N	2.03180	1.03520	2.54520
C	3.75510	1.59740	-0.17270
H	4.44690	1.71270	-1.02130
H	4.34280	1.73050	0.75130
C	0.80340	-2.86610	-0.38190
H	0.10410	-2.65140	0.43290
H	1.26430	-3.83880	-0.16030
C	3.19200	-2.10340	-0.40030
C	0.03820	-2.91250	-1.70140

H	0.68340	-3.27150	-2.51950
H	-0.78180	-3.64440	-1.59460
C	-0.72680	-1.32510	2.90850
C	3.49980	-3.27700	-1.33540
H	3.19410	-4.23030	-0.88260
H	4.58090	-3.34110	-1.51950
H	2.99000	-3.17350	-2.30130
C	5.35280	-0.75310	-0.65470
H	5.83540	0.08390	-1.17830
H	5.82360	-1.67620	-1.02040
H	5.56680	-0.65360	0.41820
C	3.55610	-0.55420	-2.41370
H	2.48640	-0.68290	-2.62590
H	4.13110	-1.25410	-3.03640
H	3.84820	0.46340	-2.70970
C	1.77040	1.89580	3.27410
C	-5.70880	-1.35420	-1.02400
C	-1.89590	-1.53660	3.74100
H	-2.64790	-2.10370	3.17190
H	-2.31790	-0.56130	4.02760
H	-1.62110	-2.09700	4.64730
B	-1.76470	2.84940	-1.98450
H	-1.75260	3.14750	-3.15270
C	1.40910	2.98950	4.15610
H	1.67870	2.74430	5.19460
H	0.32270	3.15710	4.09020
H	1.93550	3.90530	3.84750
C	3.63640	-2.44060	1.02950
B	-1.51770	4.05340	-0.74380
H	-1.29650	5.18740	-1.09700
B	-1.73990	2.14920	1.42360
H	-1.79430	2.03760	2.64130
B	-2.11690	0.65720	0.53040
H	-2.67180	-0.27400	1.09820
C	-7.03810	-0.98710	-1.47220
H	-7.12800	0.11010	-1.48800
H	-7.79090	-1.40410	-0.78570
H	-7.21060	-1.38100	-2.48540
B	-0.74130	1.38870	-1.62640
H	-0.26270	1.07020	-2.68670
B	-3.22560	2.08130	0.47880
H	-4.33200	1.84300	0.92040
B	-2.70600	1.42740	-1.23330
H	-3.47590	0.82010	-1.93750
B	-3.07830	3.17050	-0.89960
H	-4.08360	3.64640	-1.37790
B	-0.63880	3.17420	0.56940
H	0.20890	3.84280	1.13060
B	-2.40410	3.61320	0.71060
H	-2.88810	4.50690	1.37790
H	4.67310	-2.80500	1.05460

H	3.56630	-1.56600	1.69490
H	2.99120	-3.23620	1.43200
C	-1.99010	-4.41180	1.48200
H	-1.06090	-3.91390	1.78640
H	-2.43100	-4.89860	2.36720
H	-1.75140	-5.18040	0.72760
H	-3.75520	-3.80520	0.83880
N	-4.64360	-1.63200	-0.66810
Li	1.59460	-0.26420	0.91650
Li	-2.75710	-1.78860	-0.02690

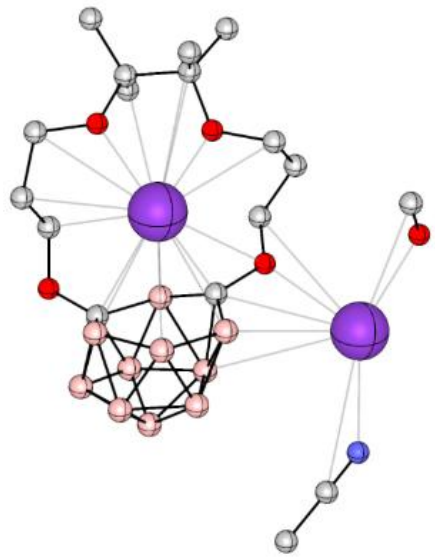

$^{mod1}K_2{}^{14C4}Cb$:

K	3.25070	2.55300	-0.46660
K	-1.09230	-0.48990	1.60800
O	1.77710	4.67450	0.21070
O	0.96020	1.19400	-0.81420
O	-3.49980	-0.96810	0.37760
O	-0.53440	-3.58740	-0.97530
O	-2.30360	1.35070	0.06520
C	-1.82820	-3.00950	-1.01430
H	-2.35080	-3.46790	-1.87120
H	-1.76010	-1.93040	-1.22950
C	1.13340	-0.10200	-0.32860
C	0.50200	-2.79420	-0.41350
C	-0.07430	1.51180	-1.75600
H	-0.77130	0.66980	-1.84690
H	0.37880	1.68480	-2.74770
C	-4.27140	0.04240	-0.31890
C	-2.66220	-3.22270	0.25280
H	-2.97340	-4.27740	0.33040
H	-2.05380	-3.02900	1.15030
C	-3.90250	-2.33590	0.26180
H	-4.47170	-2.50670	-0.66670

H	-4.56530	-2.60200	1.10410
C	-1.49640	2.53010	0.08160
H	-0.71950	2.39560	0.85530
H	-2.07010	3.42080	0.37450
C	-3.74050	1.42610	0.22230
C	-0.81990	2.74770	-1.26970
H	-1.57270	3.01400	-2.02990
H	-0.12440	3.59840	-1.17540
C	-4.31740	2.59520	-0.58890
H	-4.08340	3.55550	-0.10800
H	-5.41180	2.52810	-0.64850
H	-3.91340	2.61640	-1.60950
C	-5.76230	-0.14330	-0.00240
H	-6.15380	-1.03900	-0.50550
H	-6.35610	0.71030	-0.35620
H	-5.92750	-0.26070	1.07780
C	-4.01520	-0.08310	-1.82550
H	-2.97220	0.16600	-2.06120
H	-4.67820	0.58000	-2.39940
H	-4.21350	-1.11030	-2.16300
C	6.29780	0.36520	-0.19350
B	1.90910	-2.65570	-1.36050
H	1.94010	-3.05600	-2.49640
C	-4.06090	1.61750	1.71090
B	1.81860	-3.76670	-0.01300
H	1.75300	-4.94670	-0.25760
B	1.78070	-1.65460	1.95970
H	1.80800	-1.41950	3.16020
B	1.98440	-0.20690	0.92070
H	2.38520	0.84940	1.38680
C	6.94480	-0.92640	-0.04800
H	7.14950	-1.12240	1.01520
H	7.89090	-0.93880	-0.61030
H	6.27740	-1.71120	-0.43540
B	0.69370	-1.32450	-1.13290
H	0.16680	-1.16900	-2.20670
B	3.24910	-1.48240	1.00960
H	4.31030	-1.07200	1.42590
B	2.65290	-1.05780	-0.74890
H	3.34100	-0.42640	-1.51690
B	3.24900	-2.70280	-0.26200
H	4.30710	-3.08890	-0.70220
B	0.82850	-2.90000	1.22180
H	0.06450	-3.60850	1.84990
B	2.63290	-3.08120	1.38710
H	3.22840	-3.83570	2.12920
H	-5.12540	1.84450	1.86780
H	-3.82000	0.71690	2.29590
H	-3.47940	2.46470	2.10630
C	1.04310	4.76840	1.42970
H	1.18820	3.82960	1.98500

H	1.40280	5.60140	2.06030
H	-0.03820	4.89920	1.25010
H	1.62950	5.48590	-0.30070
N	5.76630	1.38820	-0.31200

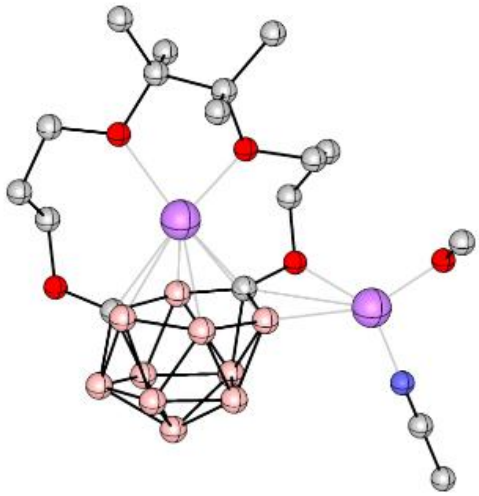

^{mod1}Li$_2$^{14C4}Cb:

O	3.50720	2.95550	0.68620
O	1.71050	0.61640	-1.03800
O	-3.22150	-0.16690	-0.07920
O	-1.28800	-3.42190	-1.05890
O	-1.31550	1.57590	0.21340
C	-2.37080	-2.62080	-1.52720
H	-2.71120	-3.09470	-2.44960
H	-2.00780	-1.62370	-1.79560
C	1.14010	-0.44460	-0.27420
C	-0.34380	-2.75950	-0.23200
C	0.80030	1.39040	-1.84610
H	-0.07150	0.78180	-2.08320
H	1.31950	1.61580	-2.77860
C	-3.60470	1.21720	-0.35900
C	-3.51590	-2.52130	-0.51440
H	-4.28490	-3.26650	-0.73170
H	-3.13480	-2.74310	0.47880
C	-4.17620	-1.16260	-0.49480
H	-4.56110	-0.91920	-1.48830
H	-5.01880	-1.17310	0.19990
C	-0.18310	2.46230	0.25110
H	0.55200	2.00140	0.90500
H	-0.44640	3.41530	0.70020
C	-2.65110	2.08780	0.52160
C	0.39780	2.67740	-1.13900
H	-0.32310	3.20050	-1.77280
H	1.27250	3.32620	-1.03720
C	-2.78860	3.57270	0.17660
H	-2.28710	4.19220	0.91840

H	-3.83720	3.86110	0.18510
H	-2.38040	3.80430	-0.80490
C	-5.06990	1.44750	0.02100
H	-5.73350	0.95420	-0.68780
H	-5.31090	2.50800	-0.00210
H	-5.28570	1.06720	1.01840
C	-3.38230	1.49260	-1.84700
H	-2.32470	1.43400	-2.09820
H	-3.75480	2.47970	-2.11990
H	-3.92310	0.76490	-2.45130
C	6.08470	0.38570	-1.00600
B	1.25350	-3.23760	-0.51460
H	1.52360	-3.90670	-1.45760
C	-2.87810	1.87780	2.01820
B	0.33910	-3.86840	0.82840
H	-0.05440	-4.98870	0.76690
B	0.36590	-1.37580	2.26230
H	0.06620	-0.82890	3.29080
B	1.44850	-0.40280	1.19920
H	2.06080	0.56010	1.58740
C	7.41200	-0.01030	-1.41900
H	7.96300	-0.38910	-0.55690
H	7.93790	0.85020	-1.83500
H	7.34250	-0.79290	-2.17580
B	0.53160	-1.62330	-1.01780
H	0.45110	-1.58730	-2.20700
B	2.03060	-1.87270	2.00000
H	2.94160	-1.66330	2.74470
B	2.25590	-1.79620	0.10250
H	3.31860	-1.60870	-0.39570
B	2.02980	-3.33080	1.01830
H	2.96140	-4.06970	1.10210
B	-0.64030	-2.49080	1.42270
H	-1.72950	-2.78780	1.81150
B	0.81820	-3.07040	2.31140
H	0.81910	-3.72050	3.31530
H	-3.81370	2.33240	2.34440
H	-2.89780	0.81790	2.27220
H	-2.06320	2.35200	2.56750
C	2.85050	3.74710	1.69220
H	2.15270	3.09460	2.21140
H	3.57770	4.13390	2.40890
H	2.30500	4.57720	1.23870
H	4.21980	3.47320	0.29020
N	5.03080	0.69690	-0.67420
Li	-1.12040	-0.36550	0.44260
Li	3.22830	1.20650	-0.00710

S9. References

[1] K. Ohta, T. Goto, H. Yamazaki, F. Pichierri, Y. Endo, *Inorg. Chem.* **2007**, *46*, 3966-3970.

[2] K. Zong, J. R. Reynolds, *J. Org. Chem.* **2001**, *66*, 6873-6882.

[3] M. Keener, C. Hunt, T. G. Carroll, V. Kampel, R. Dobrovetsky, T. W. Hayton, G. Ménard, *Nature* **2020**, *577*, 652-655.

[4] P. S. Guin, S. Das, *Int. J. Electrochem.* **2014**, *2014*, 517371.

[5] A. K. Harit, R. Das, P. Bose, *J. Found. Appl. Phys.* **2017**, *4*, 62-73.

[6] X.-Z. Feng, Z. Lin, L.-J. Yang, C. Wang, C.-l. Bai, *Talanta* **1998**, *47*, 1223-1229.

[7] S. Ramotowska, A. Ciesielska, M. Makowski, *Molecules* **2021**, *26*, 3478.

[8] J. Popovic, *ACS Phys. Chem. Au* **2022**, *2*, 490-495.

[9] D. Morales, R. E. Ruther, J. Nanda, S. Greenbaum, *Electrochim. Acta* **2019**, *304*, 239-245.

[10] K. Yoshida, M. Tsuchiya, N. Tachikawa, K. Dokko, M. Watanabe, *J. Phys. Chem. C* **2011**, *115*, 18384-18394.

[11] *Gaussian 09*, *Revision D.01*, M. J. Frisch, G. W. Trucks, H. B. Schlegel, G. E. Scuseria, M. A. Robb, J. R. Cheeseman, G. Scalmani, V. Barone, B. Mennucci, G. A. Petersson, H. Nakatsuji, M. Caricato, X. Li, H. P. Hratchian, A. F. Izmaylov, J. Bloino, G. Zheng, J. L. Sonnengerg, M. Hada, M. Ehara, K. Toyota, R. Fukuda, J. Hasegawa, M. Ishida, T. Nakajima, Y. Honda, O. Kitao, H. Nakai, T. Vreven, J. A. Montgomery Jr, J. E. Peralta, F. Ogliaro, M. Bearpark, J. J. Heyd, E. Brothers, K. N. Kudin, V. N. Staroverov, T. Keith, R. Kobayashi, J. Normand, K. Raghavachari, A. Rendell, J. C. Burant, S. S. Iyengar, J. Tomasi, M. Cossi, N. Rega, N. J. Millam, M. Klene, J. E. Knox, J. B. Cross, V. Bakken, C. Adamo, J. Jaramillo, R. Gomperts, R. E. Stratmann, O. Yazyev, A. J. Austin, R. Cammi, C. Pomelli, J. W. Ochterski, R. L. Martin, K. Morokuma, V. G. Zakrzewski, G. A. Voth, P. Salvador, J. J. Dannenberg, S. Dapprich, A. D. Daniels, Ö. Farkas, J. B. Foresman, J. V. Ortiz, J. Cioslowski, D. J. Fox, Gaussian, Inc., Wallingford CT, 2010.

[12] A. D. Becke, *J. Chem. Phys.* **1993**, *98*, 5648-5652.

[13] C. Lee, W. Yang, R. G. Parr, *Phys. Rev. B* **1988**, *37*, 785-789.

[14] J. P. Perdew, *Phys. Rev. B* **1986**, *34*, 7406-7406.

[15] A. D. Becke, *Phys. Rev. A* **1988**, *38*, 3098-3100.

[16] F. Weigend, R. Ahlrichs, *Phys. Chem. Chem. Phys.* **2005**, *7*, 3297-3305.

[17] A. V. Marenich, C. J. Cramer, D. G. Truhlar, *J. Phys. Chem. B.* **2009**, *113*, 6378-6396.
